# Tumor-killing nanoreactors fueled by tumor debris can enhance radiofrequency ablation therapy and boost antitumor immune responses

**DOI:** 10.1038/s41467-021-24604-9

**Published:** 2021-07-14

**Authors:** Zhijuan Yang, Yujie Zhu, Ziliang Dong, Wei Li, Nailin Yang, Xianwen Wang, Liangzhu Feng, Zhuang Liu

**Affiliations:** 1 Jiangsu Key Laboratory for Carbon-Based Functional Materials & Devices, Institute of Functional Nano & Soft Materials (FUNSOM), Soochow University, Suzhou, Jiangsu PR China; 2 Macao Institute of Materials Science and Engineering, Macau University of Science and Technology, Taipa, Macau, PR China

**Keywords:** Drug delivery, Organic-inorganic nanostructures

## Abstract

Radiofrequency ablation (RFA) is clinically adopted to destruct solid tumors, but is often incapable of completely ablating large tumors and those with multiple metastatic sites. Here we develop a CaCO_3_-assisted double emulsion method to encapsulate lipoxidase and hemin with poly(lactic-co-glycolic acid) (PLGA) to enhance RFA. We show the HLCaP nanoreactors (NRs) with pH-dependent catalytic capacity can continuously produce cytotoxic lipid radicals via the lipid peroxidation chain reaction using cancer cell debris as the fuel. Upon being fixed inside the residual tumors post RFA, HLCaP NRs exhibit a suppression effect on residual tumors in mice and rabbits by triggering ferroptosis. Moreover, treatment with HLCaP NRs post RFA can prime antitumor immunity to effectively suppress the growth of both residual and metastatic tumors, also in combination with immune checkpoint blockade. This work highlights that tumor-debris-fueled nanoreactors can benefit RFA by inhibiting tumor recurrence and preventing tumor metastasis.

## Abstract (web summary)

Radiofrequency ablation (RFA) is a minimally invasive tumor ablation method, however incomplete ablation and the induction of an immunosuppressive microenvironment limit its efficacy in the clinic. Here the authors design a pH-responsive lipoxidase-loaded nanoreactor, that by triggering ferroptosis and anti-tumor immunity, amplify the therapeutic benefits of RFA in preclinical models.

## Introduction

Radiofrequency ablation (RFA) is a minimally invasive tumor ablation method by utilizing heat generated from the RF probe driven by high-frequency electric pulses to locally ablate tumors^1,2^. Currently, RFA has become a first-line treatment method to replace surgery for patients with early-stage non-metastatic liver cancers in China^3,4^. However, according to the clinical experiences, RFA may show limited therapeutic efficacy to large tumors (>5 cm) due to incomplete ablation^5–7^. Moreover, it is generally difficult to adopt RFA in treating cancer patients with multiple metastatic sites (*n* > 3). To achieve improved therapeutic outcomes, the rational combinations of RFA with immune checkpoint inhibitor such as anti-cytotoxic T-lymphocyte-associated protein-4 (anti-CTLA4) or anti-programmed cell death protein 1 (anti-PD-1) against unresectable hepatocellular carcinoma have been studied under independent clinical trials^8–10^. However, it has been discovered in a recent report that the inflammation induced after incomplete RFA would even promote tumor progression and diminish the therapeutic effect of anti-PD-1 immunotherapy, via the chemokine (C-C motif) ligand 2 (CCL2)-mediated accumulation of immunosuppressive monocytes and tumor-associated macrophages inside the residual tumor mass^11^. Therefore, there is still an urgent need to develop a more efficient strategy to enhance the therapeutic benefit of RFA and thus further extend its clinical value.

Recently, amplification of tumor oxidative stress achieved via different approaches has found to be an effective strategy to directly induce cancer cell death or rationally synergize with other cancer treatments for cancer combination therapy^12–15^. Among them, ferroptosis featured in iron-mediated excessive peroxidation of polyunsaturated fatty acids (PUFAs) has recently recognized as a non-apoptotic pathway in regulating cell death and shown to be promising to eradicate those therapy-resistant cancer cells^16–20^. Apart from nonenzymatic initiation of lipid peroxidation driven by free radicals (e.g., hydroxyl groups), the family of lipoxidase (LOXs), a large catalog of nonheme iron-containing enzymes, have shown to be an alternative strategy to catalytically generate lipid hydroperoxides in lipid environment^21–24^. Such LOXs mediated lipid peroxidation has been identified to play pivotal roles in regulating the ferroptotic cell death induced by those ferroptosis inducers (e.g., imidazole keto erastin (IKE), erastin) and shown to be a potential candidate in inducing efficient ferroptosis for cancer treatment^25–28^. Considering the large amounts of PUFAs (e.g., phospholipids) in the tumor debris produced during RFA^29^, we thus hypothesize that the development of suitable formulations to induce continuous lipid peroxidation with the tumor debris as the PUFA source may be helpful to trigger ferroptosis and eradicate those residual tumor cells post RFA.

**Fig. 1:**
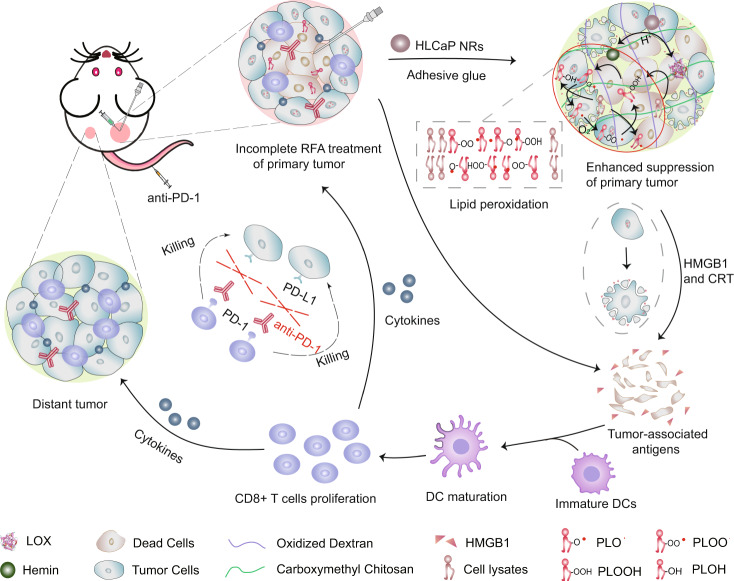
A scheme illustrating the mechanism of tumor debris fueled tumor-killing HLCaP NRs in enhancing RFA treatment and boosting antitumor immunity. Upon being fixed inside the residual tumors post incomplete RFA with adhesive glue, such HLCaP NRs in responsive to the acidic tumor microenvironment will gradually release LOX and hemin, and synergistically cause continuous lipid peroxidation from these PUFA containing phospholipids inside the tumor debris to trigger ferroptosis of residual tumor cells. Meanwhile, the released HMGB1 molecules from these ferroptotic cancer cells will recruit immature DCs to the residual tumor site and prime specific antitumor immune response featured in increased infiltration of effector T cells and secretion of effector cytokines, to further inhibit the growth of both residual tumors and metastatic (distant) tumors, especially in the combinational use of anti-PD-1 immunotherapy.

Therefore, in this study, by co-encapsulation of LOX and an iron catalyst (hemin) with PLGA via a CaCO_3_-assisted double emulsion method, we obtain a unique type of pH-responsive nanoreactors which are able to initiate continuous lipid peroxidation from the PUFA existing in tumor debris generated post RFA of tumors. By introducing sodium bicarbonate (NaHCO_3_) and calcium chlorides (CaCl_2_) to form calcium carbonate (CaCO_3_) inside the internal water phase of double emulsions, the obtained hemin and LOX co-loaded CaCO_3_-encapsulated PLGA nanoreactors (HLCaP NRs) showed higher loading efficiencies for LOX and hemin, both of which could be released in a pH-responsive manner ascribing to the pH-dependent decomposition of CaCO_3_. As a result, such HLCaP NRs would enable pH-responsive production of cytotoxic lipid radicals with these PUFAs existing in cancer cell lysates, thereby inducing immunogenic cell death (ICD). Upon being fixed inside the residual tumors post RFA by using our homemade adhesive glue, such HLCaP NRs could trigger effective lipid peroxidation and thus suppress the growth of residual tumors left post RFA treatment, on both mice and rabbits. Moreover, RFA treatment of primary tumors with the help of HLCaP NRs could result in antitumor immunity to inhibit the growth of distant tumors, especially by the combinational use of anti-PD-1 immunotherapy (Fig. 1). Therefore, this work highlights the design of effective tumor debris fueled antitumor strategy to synergistically augment the therapeutic efficacy of both conventional RFA and anti-PD-l immunotherapy, to effectively eliminate primary residual tumors with direct RFA treatment and further inhibit the tumor growth at distant metastatic sites.

## Results

### Design and characterization of tumor-killing HLCaP NRs

**Fig. 2:**
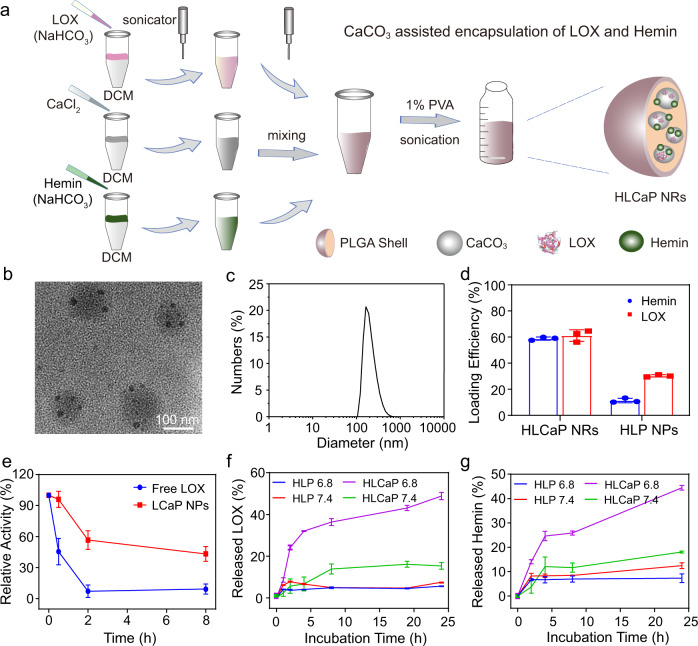
Preparation and characterization of HLCaP NRs. **a** Schematic illustration of the preparation procedure of HLCaP NRs via the CaCO_3_-assisted double emulsion process. **b** A representative TEM image of HLCaP NRs from three independent experiments. **c** Hydrodynamic diameters of HLCaP NRs determined using a Malvern Zetasizer. **d** Loading efficiencies of Hemin and LOX in HLCaP NRs and HLP NPs, the latter of which were prepared via the conventional double emulsion method without CaCO_3_. **e** The relative enzymatic activity changes of free LOX and LCaP NPs treated by protease K digestion assay. **f**, **g** Cumulative release of Hemin and LOX from HLCaP NRs and HLP NPs incubated at pH 7.4 and 6.8. Data in Fig. **d**–**g** were represented as mean ± standard error of mean (SEM), *n* = 3 biologically independent samples.

It was found that native LOX would rapidly lose its catalytic capacity in the presence of oxidized dextran, the main component of our homemade adhesive glue used in the following in vivo experiments (Supplementary Fig. 1). We thus encapsulated both LOX and hemin, an efficient iron-containing catalyst to promote the propagation of lipid peroxidation, with copolymer PLGA via the CaCO_3_ assisted double emulsion process (Fig. 2a, see details in experimental section). As visualized under the transmission electron microscopy (TEM), the obtained HLCaP NRs showed spherical morphology, and each contained several small nanoparticles with darker contrast under TEM, which should be CaCO_3_ nanocrystals formed within the internal aqueous phase of these PLGA nanoemulsions (Fig. 2b). By utilizing the calcium colorimetric assay kit, the content of CaCO_3_ in HLCaP NRs was quantified to be 18.4%. The average diameter of such HLCaP NRs was determined to be ~120 nm by using dynamic light scattering (DLS) (Fig. 2c). The loading efficiencies of LOX and hemin within these HLCaP NRs were 63.8 and 58.7%, by recording the fluorescence intensity of cyanine5.5 (Cy5.5) labeled on LOX molecules and the absorbance of hemin at 384 nm, respectively (Fig. 2d). In sharp contrast, the loading efficiencies of LOX and hemin within hemin and LOX co-loaded PLGA (HLP) nanoparticles prepared via the classical double emulsion method without introducing CaCO_3_ were only 33.4 and 11.8%, respectively. The significantly increased encapsulation efficiencies of both LOX and hemin via such CaCO_3_ assisted double emulsion method may be attributed to their strong coordination interactions between the carboxyl groups in both LOX and hemin with the newly formed CaCO_3_^30–32^.

Via the protease K digestion assay, we found that encapsulation of LOX inside those CaP nanoparticles could greatly protect the catalytic activity of LOX from being digested by protease, which widely exists in living systems (Fig. 2e). Moreover, we found that the LOX inside CaP nanoparticles persisted in higher catalytic activity than native LOX molecules after being mixed within the adhesive glue composed of 20wt.% oxidized dextran and 5wt.% carboxymethyl chitosan prepared via a previously reported method^33^ (Supplementary Fig. 2). In addition, it was found that the catalytic capacities of free LOX determined at pH 6.8 were only 67.2 and 60.5% compared to those determined at pH 7.4 and 8.0, respectively (Supplementary Fig. 3a). Therefore, we speculated that the catalytic capacity of these encapsulated LOX upon intratumoral injection would remain at a high level because our CaP nanoparticles could rapidly react with protons inside the acidic tumor microenvironment (TME) to provide LOX with a mild alkaline compartment (Supplementary Fig. 3b, c). Taken together, these results indicate that the encapsulation of LOX with CaP nanoparticles is an effective strategy to retain the catalytic capacity of LOX.

### pH-responsive cargo release and catalytic performance of HLCaP NRs

Attributing to the pH-dependent decomposition of CaCO_3_, we carefully evaluated the release behaviors of both LOX and hemin from HLCaP NRs incubated at pH 6.8, which was utilized to mimic the acidic extracellular TME of solid tumors^34^. It was found that ~48.8% of LOX and ~44.4% of hemin released from the HLCaP NRs within 24 h upon being incubated with PBS at pH 6.8, while only 15.3% of LOX and 18.1% of hemin released from HLCaP NRs after being incubated at pH 7.4 for 24 h (Fig. 2f, g). In contrast, the release rates of LOX and hemin from the counterpart HLP nanoparticles without CaCO_3_ appeared to be independent of the solution pH, indicating that the introduction of CaCO_3_ may endow pH-responsive tumor-localized release of LOX and hemin from the HLCaP NRs without imposing additional side effects to adjacent normal tissues.

**Fig. 3:**
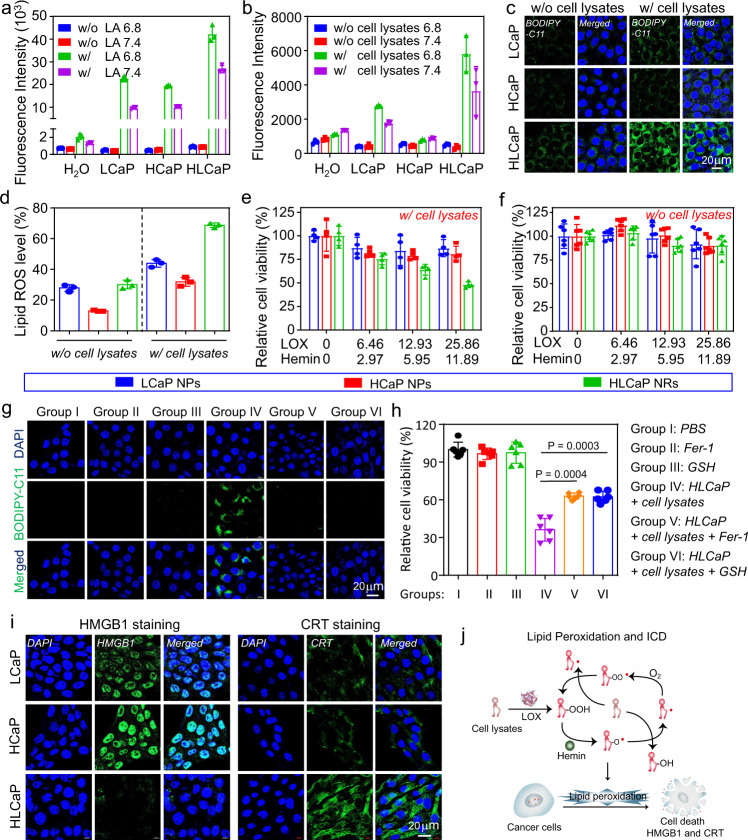
pH-responsive catalytic capacity and in vitro therapeutic efficacy of HLCaP NRs. **a**, **b** pH-responsive lipid peroxidation generation capacities of LCaP NPs, HCaP NPs, and HLCaP NRs incubated with linoleic acid (**a**) or cell lysates (**b**) at pH 6.8 and 7.4, respectively. **c**, **d** Confocal images (**c**) and flow cytometric analysis (**d**) of 4T1 cells incubated with HCaP NPs, LCaP NPs, and HLCaP NRs in the presence (w/) or absence (w/o) of cancer cell lysates, followed by being stained with lipid peroxidation probe of BODIPY-C11. **e**, **f** Relative cell viabilities of 4T1 cells incubated with HCaP NPs, LCaP NPs, and HLCaP NRs in the presence (**e**) or absence (**f**) of cancer cell lysates for 24 h before being determined by MTT assay. **g**, **h** Confocal imaging of intracellular lipid peroxidation (**g**) and relative cell viabilities (**h**) of 4T1 cells post various treatments as indicated. **i** Confocal images of 4T1 cells incubated with HCaP NPs, LCaP NPs, and HLCaP NRs in the presence of cancer cell lysates, followed by being stained with HMGB1 and CRT antibodies, respectively. **j** Schematic illustration of HLCaP NRs mediated propagation of lipid peroxidation and subsequent immunogenic cell death (ICD) induced in the presence of cell lysates. Data in Fig. **a**, **b**, **d**, **e**, **f**, and **h** were represented as mean ± SD, *n* = 3 biologically independent samples in Fig. **a**, **b**, **d**, *n* = 4 biologically independent samples in Fig. **e**, and *n* = 6 biologically independent samples in Fig. **f**, **h**. A representative image of three biologically independent samples from each group is shown in Fig. **c**, **g**, **i**. *P* values calculated by the two-tailed student’s *t*-test in Fig. **h** are indicated in the figure.

As LOX is efficient in converting linoleic acids (LAs) to its hydroperoxides (LAOOH) (Supplementary Fig. S4), we then carefully evaluated the pH-responsive catalytic performances of such HLCaP NRs in promoting lipid peroxidation with both LAs and cell lysates as the PUFA source at pH 6.8 and 7.4 detected by using the commercial lipid peroxidation sensor of BODIPY^TM^ 581/591 C11. Upon being incubated with the LA reaction solution at pH 6.8 and 7.4 for 4 h, it was shown that both LCaP and HCaP nanoparticles contributed to comparable peroxidation of LAs at both pHs, but significantly lower than that of HLCaP NRs at the same incubation conditions, respectively (LOX = 127.6 μg mL^−1^, hemin = 58.7 μg mL^−1^, BODIPY^TM^ 581/591 C11 = 1.5 μM) (Fig. 3a). However, HLP nanoparticles exhibited comparable catalytic capacity in promoting peroxidation of LAs at both pHs, but remarkably lower than that of HLCaP NRs under the same conditions (Supplementary Fig. 5a). It is worth noting that the high efficacy of HCaP nanoparticles in promoting the peroxidation of LAs should be ascribed to the auto-oxidized LAs initiated propagation of lipid peroxidation in the presence of hemin and oxygen (Supplementary Fig. 6). Moreover, the higher catalytic efficacies of these HLCaP NRs, LCaP, and HCaP nanoparticles at pH 6.8 should be attributed to the pH-responsive release of hemin and LOX. Furthermore, we found that HLCaP NRs (LOX = 127.6 μg mL^−1^, hemin = 58.7 μg mL^−1^) after being incubated with the cancer cell lysates (3 × 10^6^ cells) and BODIPY^TM^ 581/591 C11 (1.5 μM) would also contribute to more effective pH-responsive lipid peroxidation in comparison with LCaP and HCaP nanoparticles (Fig. 3b). In contrast, HLP nanoparticles only exhibited limited capacity in promoting lipid peroxidation independent of incubation pH values (Supplementary Fig. 5b). Collectively, these results demonstrated that HLCaP NRs could act as a potent pH-dependent nanoreactor to enable effective lipid peroxidation via the LOX mediated production of lipid hydroperoxide, and the subsequent propagation of lipid peroxidation in the presence of hemin and oxygen by according to these previous studies^35^.

Afterwards, we carefully explored the potency of HLCaP NRs with cancer cell lysates as the source of PUFA in inducing intracellular lipid peroxidation of cancer cells by using the BODIPY^TM^ 581/591 C11 as the lipid peroxidation probe, via both confocal microscopic observation and flow cytometric analysis. It was shown that 4T1 cells treated with HLCaP NRs (LOX = 25.86 μg mL^−1^, Hemin = 11.89 μg mL^−1^) and cell lysates (1 × 10^6^ cells) showed the most effective intracellular lipid peroxidation (Fig. 3c, d and Supplementary Fig. 7). In contrast, cells incubated with HLCaP NRs in the absence of cell lysates, and HCaP or LCaP NPs in the presence and absence of cell lysates only showed minimal lipid peroxidation. Furthermore, the potency of HLCaP NRs in inducing intracellular lipid peroxidation was confirmed by staining cells post various treatments as aforementioned with cell-permeant 2′-7′-dichlorofluorescein diacetate (DCFH-DA) as the detection probe for confocal microscopic observation and flow cytometric analysis (Supplementary Fig. 8). Therefore, these results indicate that HLCaP NRs are efficient in inducing intracellular lipid peroxidation.

### Mechanism study of HLCaP NRs induced cell death

Afterwards, the therapeutic efficacy of HLCaP NRs fueled by tumor cell lysates towards different types of cancer cells were tested by the standard methyl thiazolyl tetrazolium (MTT) assay. It was found that 51.8% of 4T1 cells were killed after being incubated with HLCaP NRs (LOX = 25.86 μg mL^−1^, hemin = 11.89 μg mL^−1^) and cell lysates (1 × 10^6^ cells) for 24 h. In contrast, the cell viabilities of these 4T1 cells treated by HCaP and LCaP NPs in the presence of cell lysates were minimally disturbed (Fig. 3e). Additionally, it was found that such HLCaP NRs showed negligible disturbance on the viability of treated 4T1 cells in the absence of cell lysates (Fig. 3f). Then, the rescuing effects of ferrostatin-1 (Fer-1) and glutathione (GSH), two efficient inhibitors of ferroptosis, on the cell death induced by HLCaP NRs plus cell lysates were carefully studied^36^. It was found that the addition of both Fer-1 and GSH could effectively suppress the production of intracellular lipid peroxides of 4T1 cells incubated with HLCaP NRs (LOX = 25.86 mg mL^−1^, hemin = 11.89 mg mL^−1^) plus cell lysates (1 × 10^6^ cells) for 6 h via confocal microscopic observation and flow cytometry analysis, using BODIPY^TM^ 581/591 C11 as the probe (Fig. 3g and supplementary Fig. 9). Consistently, we uncovered that both Fer-1 and GSH treatments could effectively reverse the cytotoxicity of HLCaP NRs plus cell lysates on 4T1 cells as well as human hepatocellular carcinoma (HepG2) cells (Fig. 3h and supplementary Fig. 10). Furthermore, we found that such HLCaP NRs also showed superior cell killing ability towards murine CT26 cells, murine B16 cells, and human MCF-7 cells in the presence of corresponding cell lysates (Supplementary Fig. 11), suggesting that such HLCaP NRs fueled by tumor cell lysate would be potent in causing cell death via the ferroptosis pathway.

It has been unfolded that ferroptosis of the cancer cell is able to promote the release of high mobility group box 1 (HMGB1) and the expression of calreticulin (CRT), two typical damage-associated molecular patterns (DAMPs), from these dying cells, and thereby elicit a series of pro-inflammatory antitumor immune responses^37–39^. As indicated by both confocal microscopic observation and flow cytometric analysis, we found that 4T1 cells treated by HLCaP NRs (LOX = 25.86 μg mL^−1^, Hemin = 11.89 μg mL^−1^) in the presence of cell lysates (1 × 10^6^ cells) for 24 h showed significantly increased release of HMGB1 from the cell nuclei and upregulated CRT expression (Fig. 3i and supplementary Figs. 12, 13), in sharp contrast to the other treatments which showed minimal influence on these DAMPs. Taken together, these results demonstrate that HLCaP NRs would gradually release a catalytic couple of LOX and hemin to cause lipid peroxidation chain reaction with cell lysates as the source of PUFA in the extracellular space. Then, the abundant lipid peroxides would efficiently damage the adjacent cell membrane, which is rich in PUFA containing lipids, and enable the propagation of lipid peroxidation inside both cell membrane and cytosol to cause cell death via the ferroptosis pathway (Fig. 3j).

### In vivo therapeutic efficacy of RFA plus HLCaP NRs fixation

**Fig. 4:**
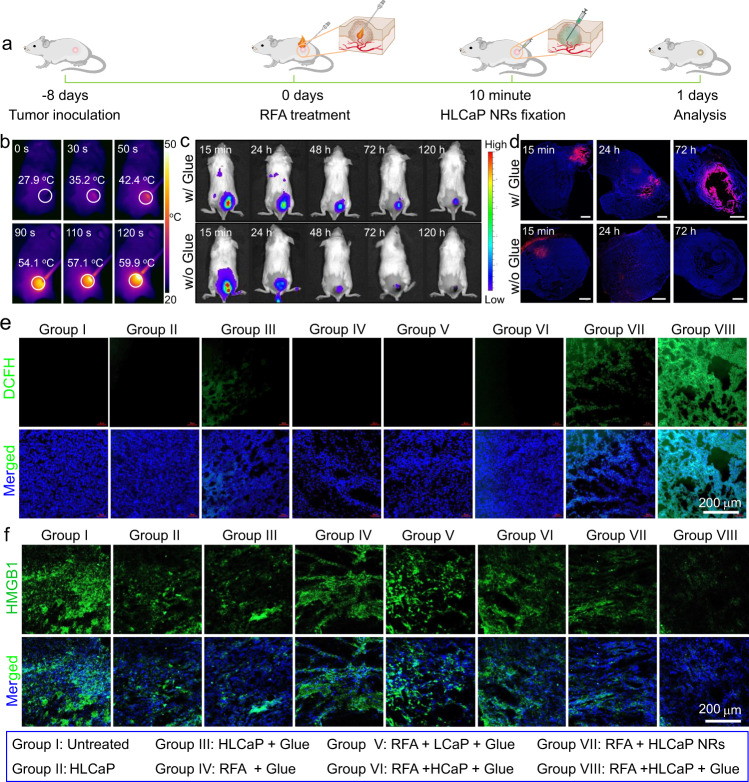
In vivo study of intratumoral lipid peroxidation and HMGB1 release post sequential RFA and HLCaP NRs fixation. **a** Schematic illustration of the experimental schedule. **b** IR thermal images of 4T1 tumor-bearing mice during RFA treatment recorded at different time points as indicated. **c** In vivo fluorescence imaging of 4T1 tumor-bearing mice with intratumoral injection of Cy5.5 labeled HLCaP NRs in the presence or absence of adhesive glue at indicated time points post RFA treatment. **d** Confocal images of tumor slices collected from 4T1 tumor-bearing mice with intratumoral injection of Cy5.5 labeled HLCaP NRs in the presence or absence of adhesive glue at indicated time points post RFA treatment. Scale bar was 1 mm. **e**, **f** Confocal images of tumor slices collected from 4T1 tumor-bearing mice after different treatments as indicated for 24 h and stained with DCFH-DA (green, **e**), as well as HMGB1 primary antibodies and corresponding Alexa 488-conjugated secondary antibodies (green, **f**). A representative image of three biologically independent animals from each group is shown in Fig. **d**–**f**.

It has been reported that clinically used RFA commonly showed limited therapeutic efficacy towards large tumors as the result of incomplete ablation-induced recurrence^11^. Herein, we carefully investigated the potency of our HLCaP NRs in inhibiting the recurrence of RFA by utilizing their capacity in inducing continuous lipid peroxidation with the tumor debris generated by RFA as the PUFA source (Fig. 4a). The partially ablated 4T1 tumor models were built by heating 4T1 tumors (~150 mm^3^) subcutaneously inoculated on Balb/c mice by using a commercial RFA system (RFA-II), with a temperature of tumor regions controlled at ~60 ^o^C for 2 min using a thermal camera (Fig. 4b). To enable sustained lipid peroxidation of tumor debris to efficiently inhibit the growth of residual tumor cells, HLCaP NRs were immobilized inside residual tumors with our homemade adhesive glue composed of 20wt.% oxidized dextran solution and 5wt.% carboxymethyl chitosan solution. By recording the fluorescence of Cy5.5 covalently labeled on the LOX of HLCaP NRs using an IVIS in vivo fluorescence imaging system, it was semiquantitatively shown that ~25% of HLCaP NRs in the presence of such adhesive glue remained at the tumor site on the fifth-day postinjection (p.i.) (Fig. 4c and supplementary Fig. 14). In contrast, only 2% of HLCaP NRs remained at the tumor site without using such adhesive glue on fifth-day p.i. Furthermore, as observed under the confocal microscopy, we found that the adhesive glue could effectively promote the retention and lateral diffusion of Cy5.5 labeled HLCaP NRs inside the residual tumor for up to 72 h (Fig. 4d), while no detectable Cy5.5 fluorescence was observed on tumor slices collected from mice at 72 h postinjection of HLCaP NRs alone. These results collectively indicate that the adhesive glue could enable long-term retention and gradual lateral diffusion of HLCaP NRs inside the residual tumor mass.

We then carefully studied the capacity of such HLCaP NRs in inducing intratumoral lipid peroxidation and ICD of cancer cells. 4T1 tumor-bearing mice (~150 mm^3^) were randomly divided into eight groups and received corresponding treatments as follows: group I, untreated; group II, HLCaP NRs only; group III, HLCaP NRs + Glue; group IV, RFA + Glue; Group V, RFA + LCaP + Glue; group VI, RFA + HCaP + Glue; group VII, RFA + HLCaP NRs; group VIII, RFA + HLCaP NRs + Glue. For the mice of group IV, V, VI, VII, and VIII, their tumors were partially ablated with the commercial RFA system (RFA-II) for 2 min as aforementioned, and intratumorally injected with different formulations as indicated. The tumors on the mice of group II and III were directly injected with varying agents as indicated. The doses of LOX, hemin, and adhesive glue for relevant groups were 425 μg, 196 μg, and 6.25 mg per mouse, respectively. At 24 h post various treatments, the tumor slices collected from each group were stained with ROS probe of DCFH-DA. As being visualized under the confocal microscopy and subsequent semiquantitative analysis, we found that tumor slices collected from group VIII showed obvious DCFH fluorescence, significantly stronger than those of group III and VII (Fig. 4e and supplementary Fig. 15). In contrast, the other tumor slices showed minimal DCFH fluorescence. Moreover, it was found that obvious DCFH and BODIPY^TM^ 581/591 C11 fluorescence signals could also be observed on tumor slices collected from the mice of group VIII at 72 h post the treatment (Supplementary Fig. 16), indicating that RFA plus intratumoral HLCaP NRs fixation could induce sustained lipid peroxidation of those residual tumor cells.

The potency of such treatments in inducing in vivo ICD of residual cancer cells were detected via the immunofluorescence staining with anti-HMGB1 and anti-CRT primary antibodies. It was found that tumor slices of group VIII showed the most efficient reduction of HMGB1 signals, which was only 34.8% compared to that of untreated groups via the semiquantitative analysis (Fig. 4f and supplementary Fig. 17). In contrast, the HMGB1 signals of the slices collected from group III and VII were quantified to be 59.4 and 50.7% via the same procedure, respectively. Consistently, it was found that the tumor slices of group VIII also exhibited the highest CRT signals (Supplementary Fig. 18), collectively demonstrating that local administration of such HLCaP NRs/glue after RFA treatment could induce effective ICD of cancer cells via initiating continuous lipid peroxidation.

**Fig. 5:**
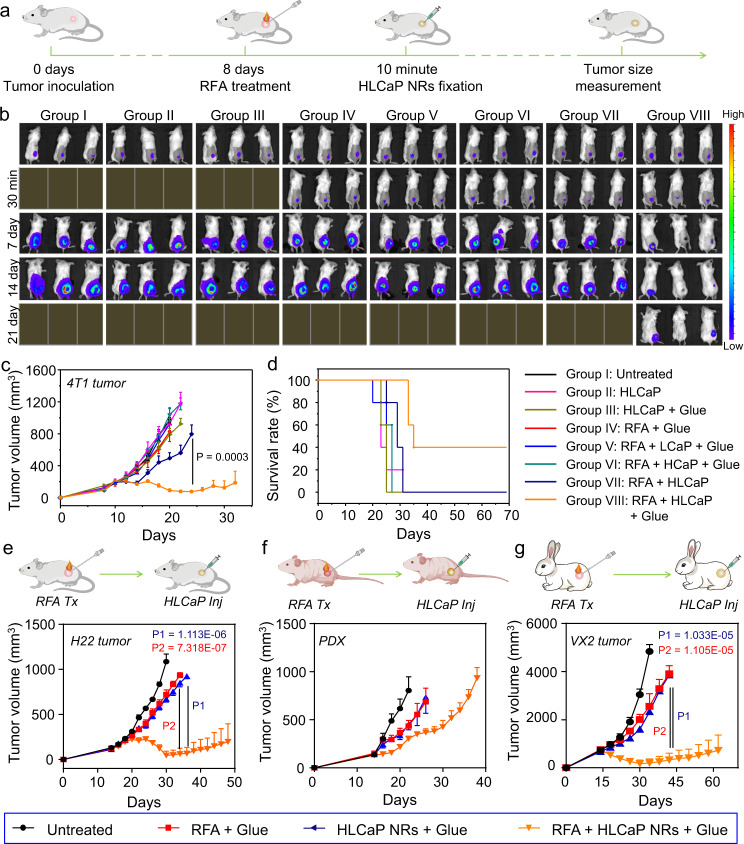
In vivo antitumor therapeutic efficacy of sequential RFA and HLCaP NRs fixation. **a** Schematic illustration of the in vivo therapeutic schedule on mouse 4T1 tumor model. **b** In vivo representative bioluminescence imaging of different groups of mice post different treatments as indicated. **c**, **d** Tumor growth curves (**c**) and corresponding mobility-free survival rate (**d**) of 4T1 tumor-bearing mice post different treatments as indicated. The mice were set as dead when their tumor volume was larger than 1000 mm^3^. **e**–**g** Schematic illustrations and corresponding tumor growth curves of murine H22 tumors (**e**), human liver cancer PDX tumors (**f**), and rabbit VX2 tumors (**g**) post different treatments as indicated. Data of Fig. **b**, **e**–**g** were represented as mean ± SEM, *n* = 5 biologically independent animals in Fig. **c**–**f**, *n* = 4 biologically independent rabbits in Fig. **g**. *P* values calculated by the two-tailed student’s *t*-test are indicated in the figure.

To evaluate the therapeutic efficacy of RFA and subsequent HLCaP NRs fixation, a total of 40 mice bearing luciferase-transfected 4T1 tumors were randomly divided into eight groups and treated as aforementioned (Fig. 5a). By recording the bioluminescence signals, it was semiquantitatively determined that ~45% tumor mass was left undestroyed after RFA treatment (2 min, Fig. 5b and supplementary Fig. 19), and the therapeutic regimen for the mice of group VIII showed the most effective tumor inhibitory effect. Furthermore, it was found that two of five mice in this group were cured without obvious recurrence as observed for up to 70 days (Fig. 5c, d). In marked contrast, the other treatments showed negligible influence on tumor growth or animal survival. Moreover, as observed via the hematoxylin and eosin (H&E) staining, we found that the tumor slices collected from the mice of group VIII showed the most severe histological damage, while the tumor slices of the other RFA related treatments of group IV, V, VI, and VII only showed moderate damages (Supplementary Fig. 20). Taken together, these results demonstrate that intratumoral fixation of HLCaP NRs could significantly improve the treatment outcomes of conventional RFA by inducing continuous lipid peroxidation of those residual tumors cells.

Considering RFA is most commonly applied for the treatment of non-metastatic liver cancers in the clinic, the therapeutic potency of RFA plus sequential intratumoral HLCaP NRs fixation was further confirmed on both murine hepatocellular carcinoma (H22) tumors on Balb/c mice and patient-derived xenografts (PDX) of hepatocellular carcinoma on Balb/c nude mice. When their tumor volume reached ~150 mm^3^, both H22 and PDX bearing mice were randomly divided into four groups (*n* = 5) and received the following treatments: group I, Untreated; group II, RFA + Glue; group III, HLCaP NRs + Glue; group IV, RFA + HLCaP NRs + Glue. These mice received the same treatments as aforementioned on day 14. As expected, it was found that the treatment of RFA plus HLCaP NRs fixation (group IV) exhibited the most effective suppression effects on the growth of both H22 and PDX tumors, while the other two treatments only slightly delayed the tumor growth (Fig. 5e, f). However, unlike four of five H22 tumors that were absolutely eradicated with no obvious tumor recurrence observed within 90 days, all PDX bearing nude mice in group IV died within 42 days (Supplementary Fig. 21a, b), likely owing to the deficient immune system of these nude mice.

We further evaluated the therapeutic potency of such RFA plus sequential intratumoral HLCaP NRs fixation using a larger animal tumor model, VX2 tumors subcutaneously inoculated on New Zealand rabbits (Fig. 5g). When their tumor volume reached ~700 mm^3^, these VX2 tumor-bearing rabbits were randomly divided into four groups (*n* = 4) and received the same treatments as depicted above for treating H22 and PDX tumors. For the rabbits of group II and group IV, their tumors were partially ablated (~50% tumor mass left estimated by naked eyes) with the aforementioned commercial RFA system for 4 min. The injection doses of LOX, hemin, and adhesive glue for relevant groups were 5, 1.74, and 62.5 mg per rabbit, respectively. As expected, it was found that the treatment of RFA plus HLCaP NRs fixation (group IV) showed the most effective inhibitory effect on VX2 tumor growth with a median survival time of 72 days, and two of four rabbits in this group was cured without obvious recurrence observed for up to 90 days. In sharp contrast, the treatments of group II (RFA + Glue) and group III (HLCaP NRs + Glue) only partially suppressed the tumor growth with their median survival times determined to be 44 and 46 days, respectively, while that was only 34 days for the untreated group (Fig. 5g and supplementary Fig. 21c). Taken together, these results further confirm the high efficacy of such intratumoral fixation of HLCaP NRs in enhancing the therapeutic efficacy of RFA, particularly for tumor models grown on animals with competent immune systems.

### HLCaP NRs boosted antitumor immunity enabling combinational cancer treatment

**Fig. 6:**
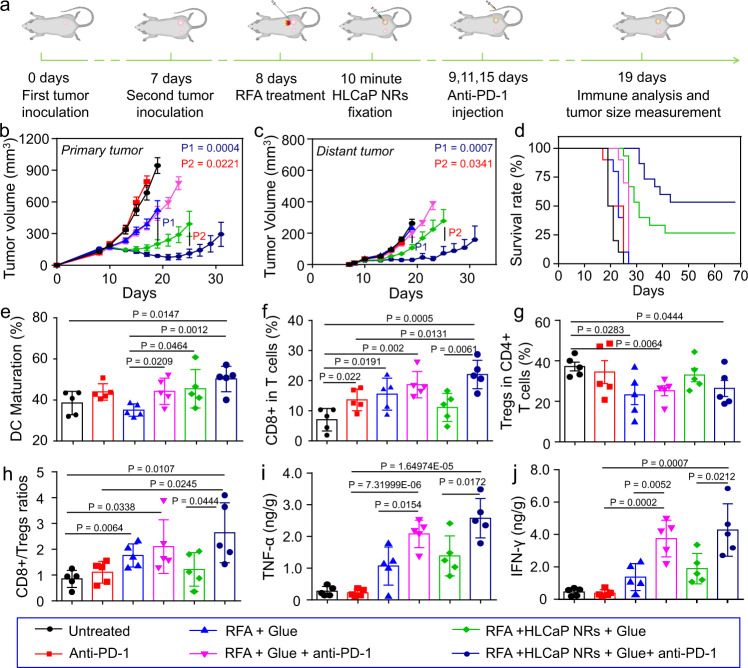
In vivo antitumor study and corresponding immune mechanism study of combined RFA, HLCaP NRs fixation, and anti-PD-1 immunotherapy. **a** Schematic illustration of the inoculation of the bilateral tumor model for in vivo antitumor and immune mechanism studies. **b**–**d** Tumor growth curves of primary (**b**) and distant tumors (**c**), as well as corresponding mobility-free survival rate (**d**) of mice with bilateral tumor models post different treatments as indicated. The mouse was set as dead when its tumor volume was larger than 1000 mm^3^. **e** DC maturation status in the drain lymph nodes adjacent to the primary tumors post various treatments as indicated. **f**–**h** The frequencies of CD3^+^CD8^+^ T cells (**f**), and CD3^+^CD4^+^FoxP3^+^ Tregs (**g**), as well as their ratios (**h**) inside the distant tumors post various treatments as indicated. **i**, **j** The secretion levels of TNF-α and IFN-γ inside the distant tumors post various treatments as indicated. Data in Fig. **b**, **c** were represented as mean ± SEM, *n* = 10 or 15 biologically independent animals, data in Fig. **e**–**j** were represented as mean ± SD, *n* = 5 biologically independent animals. *P* values calculated by the two-tailed student’s *t*-test are indicated in the figure.

Motivated by the high efficacy of HLCaP NRs together with RFA treatment in promoting HMGB1 release and CRT expression (Fig. 4f and supplementary Fig. 18), which are able to activate the host’s immune system by promoting the migration and maturation of dendritic cells (DCs)^40–42^, we thus hypothesized that such a treatment strategy may be able to trigger tumor-specific immune responses. Therefore, we then combined such sequential RFA and intratumoral HLCaP NRs fixation with anti-PD-1 immunotherapy, which can further enhance the antitumor potency of cytotoxic T cells that play a central role in the specific antitumor immune responses (Fig. 6a). Mice with two 4T1 tumors on both sides of each mouse were randomly divided into six groups (*n* = 10 or 15) and received corresponding treatments under the same dosages as abovementioned in Fig. 5b apart from some groups of mice were intravenously injected with anti-PD-1 antibody (20 μg per mouse) at day 9, 11, 15. By measuring the tumor sizes, we found that RFA plus sequential HLCaP NRs fixation could not only effectively inhibit the growth of residual primary tumors as those shown above (Fig. 5b, f), but also more efficiently suppress the growth of distant tumors, in comparison to those with their primary tumors treated by bare RFA treatment (Fig. 6b, c and Supplementary Fig. 22). Furthermore, we found that the RFA plus sequential HLCaP NRs fixation could synergize with anti-PD-1 to more effectively suppress the growth of both residual primary and distant tumors, while the bare RFA treatment showed negligible influence on the therapeutic efficacy of anti-PD-1 immunotherapy (RFA + anti-PD-1 injection). As the result, 8 of 15 mice treated by RFA plus sequential HLCaP NRs fixation and anti-PD-1 injection and 4 of 15 mice treated by sequential RFA and HLCaP NRs fixation were cured with no obvious recurrence observed within 68 days. In sharp contrast, the median survival time of mice treated by anti-PD-1 injection alone, RFA alone, and RFA + anti-PD-1 injection were 22, 24, and 28 days, respectively, while that for the mice received no treatments was 20 days (Fig. 6d). Collectively, these results demonstrate that the combination treatment of RFA and HLCaP NRs fixation could not only effectively inhibit the growth of residual primary tumors, but also suppress the growth of distant tumors, the mimic of metastatic tumors. Meanwhile, together with anti-PD-1 immunotherapy, the therapeutic efficacies of such combination therapy towards both residual primary and abscopal distant tumors were further improved.

We then carefully investigated the mechanism underlying the high efficacy of such HLCaP NRs in sensitizing both RFA treatment and anti-PD-1 immunotherapy by analyzing their effects on the immune system at 4 days post last injection of anti-PD-1. Consistent with their capacities in inducing HMGB1 release and CRT expression (Fig. 4f and Supplementary Fig. 18), the treatments of RFA plus sequential HLCaP NRs fixation, regardless of anti-PD-1 injection, could significantly promote the maturation of DCs inside the lymph nodes adjacent to the primary tumors (Fig. 6e and Supplementary, Fig. 23). Notably, we found that the combination treatment of RFA plus sequential HLCaP NRs fixation and anti-PD-1 injection could significantly promote the frequencies of tumor-infiltrating CD3^+^CD8^+^ and CD3^+^CD4^+^ T cells (Fig. 6f and Supplementary Fig. 24) inside the distant tumors, but only slightly affected the intratumoral frequency of immunosuppressive regulatory T cells (Tregs) (Fig. 6g and Supplementary Fig. 25). Moreover, such combination treatment resulted in remarkably increased CD3^+^CD8^+^/Tregs ratios (Fig. 6h), an important sign of activated antitumor immunity^43^. Moreover, the secretion levels of cytotoxic cytokines including both tumor necrosis factor alpha (TNF-α) and interferon gamma (IFN-γ) inside the distant tumors post the combination treatment were also significantly increased (Fig. 6i, j). Taken together, these results indicate that the combination treatment of sequential RFA, HLCaP NRs fixation, and anti-PD-1 injection is the most effective in priming the host’s antitumor immunity.

## Discussion

In this study, we prepared a type of tumor debris fueled tumor-killing nanoreactors by encapsulating both LOXs and hemin with PLGA via our developed CaCO_3_ assisted double emulsion process to work as the immunogenic adjuvant nanomedicine for both RFA treatment and ICB immunotherapy. The introduction of CaCO_3_ could endow highly effective encapsulation of payloads in comparison with those conventional PLGA nanoparticles without CaCO_3_. In addition, the introduction of CaCO_3_ could provide a mild alkaline condition to avoid the tumor acidity-induced deactivation of LOX upon intratumoral administration. Further, it was found that such HLCaP NRs could effectively neutralize the acidic residual tumor mass (Supplementary Fig. 3), which thus may be able to reverse the immunosuppressive TME and thus benefit ICB immunotherapy according to those previous reports^14,44^. Meanwhile, the obtained HLCaP NRs featured with the pH-dependent release profile would constraint both LOX and hemin in a localized space together with adhesive glue to enable sustained lipid peroxidation with PUFA containing tumor debris as the fuel, thereby induce effective ICD of tumor cells with increased release of HMGB1 and expression of CRT.

Upon being fixed inside the residual tumor post RFA treatment with our homemade adhesive glue, such HLCaP NRs would utilize RFA-generated tumor debris as the fuel to efficiently inhibit the growth of the residual tumors by inducing continuous lipid peroxidation, as evidenced in the treatment of 4T1 and H22 murine tumors, VX2 rabbit tumors, as well as PDX tumors on Balb/c nude mice. In the meanwhile, HLCaP NRs mediated DAMPs exposure post RFA treatment would prime the host’s specific antitumor immunity by promoting effective maturation of DCs, infiltration of effector CD8^+^ T cells, and secretion of cytotoxic cytokines. Together with anti-PD-1 immunotherapy, such RFA treatment and sequential HLCaP NRs fixation would efficiently suppress the growth of both residual primary tumors and distant metastatic tumors. Moreover, such tumor-localized fixation of HLCaP NRs with adhesive glue could retain them at the tumor site without imposing potential side effects to both adjacent and distant tissues.

In summary, this study highlights innovative tumor debris fueled antitumor strategy by utilizing the tumor-killing HLCaP NRs prepared via our CaCO_3_-assisted double emulsion process. Such HLCaP NRs with the capability in effectively inducing lipid peroxidation from tumor debris could not only enhance the therapeutic outcome of RFA, but also act as an immunogenic nanomedicine to enable the synergistic combination of RFA with ICB immunotherapy. Given that the full biocompatibility of various components in those nanoparticles, such HLCaP NRs hold great promises for future clinical translation. Moreover, considering the fact that diverse cancer treatments (e.g., radiotherapy, chemotherapy, microwave ablation) can also produce a large amount of PUFA containing tumor debris, it is speculated that such HLCaP NRs upon intratumoral fixation would be able to synergize with various types of cancer treatment methods in future clinical practices.

## Methods

### Chemicals and reagents

LOX, hemin, poly (d,l-lactic-co-glycolic acid) (PLGA), and polyvinyl alcohol (PVA) were obtained from Sigma-Aldrich. Dichloromethane (DCM), sodium bicarbonate (NaHCO_3_), and calcium chloride (CaCl_2_) were obtained from Sinopharm Chemical Reagent Co. Anti-HMGB1 antibody (catalog: 70-ab40050-100) was obtained from MultiSciences. Anti-CRT antibody (catalog: ab2907) was obtained from Abcam. Alexa 488-conjugated secondary antibody (catalog: 111-545-003) was obtained from Jackson. Antibodies for flow cytometry assays including anti-CD3-FITC (Biolegend, clone 17A2, catalog: 100204), anti-CD4-APC (Biolegend, clone GK1.5, catalog: 100412), anti-CD8-PE (Biolegend, clone 53-6.7, catalog: 100708), and anti-Foxp3-PE (Biolegend, clone MF-14, catalog: 126404), anti-CD11c-FITC (Biolegend, clone N418, catalog: 117306), anti-CD80-PE (Biolegend, clone 16-10A1, catalog: 104708), and anti-CD86-APC (Biolegend, clone GL-1, catalog: 105012) were obtained from Biolegend or eBioscience as indicated and diluted at 1:300 for cell staining. Anti-PD-1 (catalog: BE0146) was purchased from BioXcell.

### Preparation and characterization of HLCaP NRs

HLCaP NRs were synthesized via a modified double emulsion process^31,45^. In brief, LOX and hemin were firstly dissolved in NaHCO_3_ (0.625 M) at concentrations of 16 mg mL^−1^ and 8 mg mL^−1^, respectively, while PLGA was dissolved in DCM at 13.3 mg mL^−1^. Then, hemin and LOX emulsions were obtained by combining 125 μL of as-prepared hemin solution or LOX solution with 375 μL PLGA solution followed by sonication using a probe sonicator (40 kHz) for 5 min. CaCl_2_ emulsion was obtained by combining 250 μL of CaCl_2_ solution (1.25 M) with 750 μL PLGA solution followed by being sonicated under the aforementioned parameters. After that, these three emulsions were combined together and sonicated under the aforementioned parameters for 5 min to obtain HLCaP emulsion, which was then added dropwisely to 3 mL 1wt.% PVA aqueous solution under the sonication using a water bath sonicator for 5 min. After being stirred at room temperature overnight for complete evaporation of DCM, such solutions were sequentially washed three times with 18.2 Ω cm^−1^ pure water via centrifugation (21,000x*g*, 10 min) to remove unloaded LOX and hemin, and then centrifuged at 900x*g* for 3 min to remove large aggregates. The obtained HLCaP NRs were stored at 4 ^o^C for further experiments.

Cy5.5 labeled LOX was used for the preparation of Cy5.5 labeled HLCaP nanoreactors by following the aforementioned procedure. HCaP, LCaP, and HLP nanoparticles were prepared by following the aforementioned procedures without introducing corresponding components.

The morphology of such HLCaP NRs was observed under an FEI Tecnai F20 TEM. The DLS size distribution of HLCaP NRs was measured by using a Malvern Zetasizer (Nano-ZS90).

### pH-responsive release of LOX and hemin from HLCaP NRs

To avoid the interference of light-absorbing hemin on the quantification of LOX release profiles, the pH-responsive release profiles of LOX and hemin were obtained by quantifying the amounts of released LOX and hemin from the LCaP and HCaP nanoparticles, respectively. In brief, the as-prepared Cy5.5 labeled LCaP nanoparticles (LOX = 0.638 mg mL^−1^) and HCaP nanoparticles (hemin = 0.587 mg mL^−1^) were incubated with PBS at pH 6.8 or 7.4 and agitated at room temperature. At designated time intervals, the released LOX and hemin were collected by centrifugation (21,000x*g*, 10 min) to measure their concentrations.

### Catalytic activity of HLCaP NRs

To avoid the interference of light-absorbing hemin on the quantification of catalytic activity of LOX, LCaP nanoparticles were used to quantify the catalytic activity of LOX by detecting the formation of LA hydroperoxide according to the previously reported protocol^46,47^. The reaction substrate was firstly prepared by dissolving 40 μL LA with 100 μL 5wt.% NaOH solution followed by being diluted with 10 mL H_2_O, and then mixed with 40 mL phosphate buffers (PB) solution at pH 8.0 containing 50 μL tween-20. To evaluate the pH-responsive catalytic activity of HLCaP NRs, the as-prepared LCaP nanoparticles, HCaP nanoparticles, and HLCaP NRs (LOX = 127.6 μg mL^−1^, hemin = 58.7 μg mL^−1^) were mixed with LA (450 μg mL^−1^) or cancer cell lysates (3 × 10^6^ cells mL^−1^) at pH 6.8 or 7.4 for 4 h. Then, the BODIPY-C11 probe (1.5 μM) was added and incubated for 30 min before the samples’ fluorescence intensities were recorded using a microreader (Ex. = 488 nm, Em. = 530 nm). Cancer cell lysates were obtained via three repeated freeze-thaw cycles.

### Preparation and characterization of adhesive glue

The oxidized dextran was prepared by simply mixing dextran (59 mg mL^−1^) with sodium periodate (121.8 mg mL^−1^) in pure water, followed by being stirred in dark at room temperature according to the previously reported method. Twenty-four hours later, the reaction mixture was collected and purified by using a dialysis tube (molecular weight cut-off = 3500 Da) against water for 48 h before lyophilization. The successful synthesis of oxidized dextran was confirmed using the FT-IR spectrometer (FTIR) and proton nuclear magnetic resonance (^1^H NMR).

The adhesive glue was prepared by simply mixing 20wt.% oxidized dextran solution and 5wt.% commercial carboxymethyl chitosan solution^33^. The gelation process of such adhesive glue was monitored using a rotational rheometer (Haake Rheo Stress 6000, Germany, PP20 H, gap set at 0.5 mm, frequency = 1 Hz) at 37 °C, and its morphology after lyophilization was observed using the scanning electron microscopy (SEM, Zeiss Supra 55).

### Cell Experiments

Murine 4T1 breast cancer cells (SCSP-5056), murine CT26 colon cancer cells (TCM37), human HepG2 hepatocellular carcinoma cells (SCSP-510), murine B16 melanoma cells (TCM-2), and human MCF-7 breast cancer cells (SCSP-531) were obtained from the Cell Bank, Shanghai Institutes for Biological Sciences, Chinese Academy of Sciences.. Luciferase-transfected 4T1 cells (Luc-4T1) was obtained from PerkinElmer Co. as a gift, murine H22 hepatocellular carcinoma cells (ZQ0109) was obtained from Shanghai Zhongqiao Xinzhou Biological Technology Co., Ltd. Rabbit VX2 liver cancer cells (MZ-0769) was obtained from Ningbo Mingzhou Biological Technology Co., Ltd. All the cell lines were authenticated and checked for mycoplasma contamination by their suppliers. No mycoplasma contamination was found before use.

To evaluate the capacity of such HLCaP NRs in inducing intracellular lipid peroxidation, both 4T1 cells pre-seeded in the 12-well plate (1 × 10^5^ cells per well) were incubated with LCaP nanoparticles, HCaP nanoparticles, or HLCaP NRs (LOX = 25.86 μg mL^−1^, hemin = 11.89 μg mL^−1^) in the presence or absence of cell lysates (1 × 10^6^ dead cells per wells) for 6 h. Later, these treated cells were washed with PBS and incubated in the fresh medium containing BODIPY-C11 dye (1.5 μM) for 30 min before being washed with PBS, fixed with 4wt.% paraformaldehyde solution, stained with 4,6-diamino-2-phenyl indole (DAPI, 1 μg mL^−1^) and imaged by using the confocal microscopy (Zeiss, LSM 800). In addition, these 4T1 cells post various treatments and BODIPY-C11 staining were evaluated using a flow cytometer (BD, Accuri^TM^ C6 Plus). Moreover, these 4T1 cells post various treatments as abovementioned for 4 h were incubated in the fresh medium containing DCFH-DA (20 μM) for 30 min before being analyzed via the confocal microscopic observation and flow cytometric analysis as aforementioned.

To evaluate the rescuing effects of Fer-1 and GSH, both 4T1 and HepG2 cells pre-seeded in the 12-well plate (1 × 10^5^ cells per well) were incubated with HLCaP NRs (LOX = 25.86 μg mL^−1^, hemin = 11.89 μg mL^−1^) with cell lysates (1 × 10^6^ dead cells per wells) in the presence and absence of Fer-1 (10 μΜ) and GSH (1 mM) for 6 h before being stained and subjected to confocal microscopic observation and flow cytometric analysis as aforementioned.

To evaluate the HMGB1 release profile, 4T1 cells post various treatments as abovementioned for 24 h were washed twice with PBS, fixed in 4wt.% paraformaldehyde solution for 20 min, permeabilized with 0.1wt.% Triton X-100 for 10 min, blocked with 5% FBS for 30 min, stained with primary anti-HMGB1 antibody (dilution: 1:1000) for 1 h, and Alexa 488-conjugated secondary antibody (dilution: 1:500) for 30 min by following the manufacturer’s procedure. Later, these cells were counterstained with DAPI for 10 min and observed by using confocal microscopy as aforementioned. In addition, the HMGB1 release profiles of those cells post various treatments were evaluated by flow cytometry after being stained as abovementioned.

To evaluate the CRT expression profile, 4T1 cells pre-seeded in 12-well plates (1 × 10^5^ cells per well) received the same treatments as aforementioned before being sequentially stained with the primary anti-CRT antibody (dilution: 1:1000) for 1 h, Alexa 488-conjugated secondary antibody (dilution: 1:500) for 30 min and DAPI for 10 min. After that, these counterstained cells were subjected to confocal microscopic observation and flow cytometric analysis.

To evaluate the capacity of such HLCaP NRs in inducing cell death, 4T1 cells, HepG2 cells, MCF-7 cells, B16 cells, and CT26 cells pre-seeded in 12-well plates (1 × 10^5^ cells per well) were incubated with LCaP NPs, HCaP NPs, and HLCaP NRs in the presence or absence of corresponding cell lysates (1 × 10^6^ dead cells per well) for 24 h before their cell viabilities determined using the standard MTT assay. To evaluate the rescuing effects of Fer-1 and GSH in inducing cell death, both 4T1 and HepG2 cells pre-seeded in 12-well plates (1 × 10^5^ cells per well) were incubated with HLCaP NRs plus cell lysates (1 × 10^6^ dead cells per well) in the presence and absence of Fer-1 (10 μΜ) and GSH (1 mM) for 24 h before their cell viabilities determined using the standard MTT assay as aforementioned.

### Animal experiments

Female Balb/c mice (6–8 weeks), female Balb/c nude mice (6–8 weeks), and SPF New Zealand white rabbits (3–5 months) were purchased from Laboratory Animal Center of Soochow University, and used by following the protocols approved by Laboratory Animal Center of Soochow University. To inoculate the subcutaneous 4T1 tumor model, Luc-4T1 cells or 4T1 cells (2 × 10^6^) suspended in 50 μL PBS were subcutaneously injected to the back of each mouse. To inoculate the subcutaneous H22 tumor, H22 cells (2 × 10^6^) suspended in 50 μL PBS were subcutaneously injected into the back of each mouse. Human hepatocellular carcinoma tissue with a thick trabecular type was resected from the patient in Eastern Hepatobiliary Surgery Hospital, Shanghai, China and maintained under good clinical practice approved by the China Food and Drug Administration (CFDA). Written informed consent was provided by the patient, and the Institute Review Board (IRB) of Eastern Hepatobiliary Surgery Hospital approved this experiment. To inoculate the subcutaneous PDX tumors, these tissues were cut into small pieces (~3 mm) and then implanted to the flank of each nude mouse. To inoculate the rabbit tumor model, VX_2_ liver cancer cells (2 × 10^6^) suspended in 50 μL PBS were firstly subcutaneously injected to the back of nude mice. When their volume reached ~800 mm^3^, these VX2 tumors were collected and cut into small pieces before being subcutaneously injected to the back of each rabbit for the inoculation of VX2 rabbit tumors.

The commercial RFA system (RFA-II) and corresponding 2.2-mm single needles (RFA-0115) working at a temperature-power control mode, a 200-W generator, and a 330-kHz operating frequency were purchased from Beijing Blade Opto-Electronic Technology Development CL., Ltd. To obtain partially ablated tumors (~50% tumor mass left undestroyed) for corresponding experiments, 4T1, H22, human PDX tumors (~150 mm^3^), and VX2 tumors (~700 mm^3^) were treated by the RFA system at a targeted temperature of 80 ^o^C, an output energy limit of 85% and a heating rate of 20 ^o^C min^−1^. The tumor surface temperature as recorded using an IR thermal camera (Fortric 225) was controlled to be below 60 ^o^C by repeatedly turning on/off the system, and the total heating durations of murine tumors and rabbit tumors were 2 and 4 min, respectively.

During tumor inoculation and RFA treatment, mice and rabbits were anesthetized by intraperitoneal injection with pentobarbital sodium solution (75 mg kg^−1^) and intramuscular injection with xylazine solution (2 mg kg^−1^), respectively.

### Intratumoral retention of HLCaP NRs post RFA

To evaluate the effect of our homemade adhesive glue on the intratumoral retention of the as-prepared HLCaP NRs, the Cy5.5 labeled HLCaP NRs with or without the adhesive glue were injected into the residual tumor mass via the hole left via the incomplete RFA as abovementioned. Then, these treated mice were subjected to an IVIS in vivo fluorescence imaging system at different time intervals post the RFA treatment to record the remained Cy5.5 fluorescence intensity (Ex. = 676 nm, Em. = 705 nm). In addition, at 15 min, 24 h, and 72 h postinjection, one mouse was randomly picked out from each group, and sacrificed with their tumors collected and cryosectioned for confocal microscopic observation.

### In vivo cancer combination therapy

Luc-4T1 tumor-bearing Balb/c mice (~150 mm^3^) were randomly divided into eight groups (*n* = 5) and received the following treatments: group I, Untreated; group II, HLCaP NRs; Group III, HLCaP NRs + Glue; group IV, RFA + Glue; group V, RFA + LCaP NPs + Glue; group VI, RFA + HCaP NPs + Glue; group VII, RFA + HLCaP NRs; Group VIII, RFA + HLCaP NRs + Glue. For RFA treatments, the RF probe presterilized with 75% ethanol was inserted into the tumor on each mouse of related groups, and heated under the parameters as abovementioned. Ten minutes later, various agents were injected into residual tumor masses or intact tumors as abovementioned, and the injection doses of LOX and hemin were 425 μg per mouse and 196 μg per mouse, respectively. The injection volume of adhesive glue was 50 μL. The tumor volume (V) of each mouse was monitored by recording the length (L) and width (W) of each tumor using the digital caliper every other day, and calculated by following the equation of V = L*W*W/2. The bioluminescence intensity of each mouse before and after various treatments was recorded using the IVIS Spectrum imaging system. H22 tumor-bearing mice and PDX bearing mice received the same treatments as aforementioned.

To evaluate the intratumoral lipid peroxidation levels post various treatments, tumor-bearing mice were sacrificed at 24 and 72 h post various treatments as aforementioned, and their tumors were collected, cryosectioned, stained with DCFH-DA (20 μM) or BODIPY-C11 (1.5 μM), and DAPI before microscopic observation. Meanwhile, these tumor slices were also stained with anti-HMGB1 and anti-CRT primary antibodies, and corresponding secondary antibodies as aforementioned staining procedure to evaluate the HMGB1 release and CRT expression profiles. Additionally, these tumor slices were also analyzed via H&E staining.

To further verify the therapeutic potency of our strategies, a total of 16 VX2 tumor-bearing rabbits (~700 mm^3^) were randomly divided into four groups (*n* = 4 each group) and received different treatments as follows: group I, Untreated; group II, HLCaP NRs; group III, RFA + Glue; group IV, RFA + HLCaP NRs + Glue. For RFA treatments, the tumors on the mice of related groups were partially ablated as abovementioned. Ten minutes later, bare adhesive glue or HLCaP NRs mixed with adhesive glue were injected into the residual tumors of related groups. The doses of LOX and hemin were 4.25 and 1.96 mg, respectively, and the injection volume of adhesive glue was 500 μL. The tumor volume (V) of each rabbit was monitored by recording the length (L) and width (W) of each tumor using the digital caliper every other day.

### In vivo combined immunotherapy and mechanism study

The bilateral tumor model was built by subcutaneously injecting 4T1 cells (2 × 10^6^) suspended in 50 μL PBS into the right and left flank of each mouse as the primary or distant tumors at day 0 and day 7, respectively. On day 8, these bilateral 4T1 tumor-bearing Balb/c mice were randomly divided into six groups and treated as follows: group I, untreated; group II, anti-PD-1 injection; group III, RFA + Glue; group IV, RFA + Glue + anti-PD-1 injection; group V, RFA + HLCaP NRs + Glue; group VI, RFA + HLCaP NRs + Glue + anti-PD-1 injection. These primary tumors (~150 mm^3^) on the right side of corresponding groups of mice were partially ablated and injected with HLCaP NRs and adhesive glue under the aforementioned experimental conditions. Anti-PD-1 (20 μg per mouse) was intravenously injected into the mice of corresponding groups on days 9, 11, and 15. The tumor volumes (V) of primary and distant tumors on each mouse were monitored every other day.

To study the immune mechanism underlying such effective combined immunotherapy, another batch of bilateral tumor models was treated as aforementioned (*n* = 5). At 4 days post the last injection of anti-PD-1 (day 15), these mice were sacrificed with their lymph nodes close to the primary tumors and the distant tumors collected, homogenized, and enzymatically digested to produce single-cell suspensions according to the well-established protocol^48^ for following antibodies labeling. Then, the DCs (CD80^+^CD86^+^) inside the lymph nodes, and CD8^+^ T cells (CD3^+^CD4^-^CD8^+^) and Tregs (CD3^+^CD4^+^Foxp3^+^) inside the distant tumors were stained with corresponding commercial fluorophore-labeled antibodies by following the manufacturers’ procedure and analyzed via the flow cytometry. The supernatants of these tumors post homogenization were collected with the concentrations of TNF-α (Invitrogen, catalog: 88-7324-88) and IFN-γ (Invitrogen, catalog: 88-7314-88) determined with corresponding ELISA kits according to vendors’ instructions.

### Reporting summary

Further information on research design is available in the Nature Research Reporting Summary linked to this article.

## Supplementary information

Supplementary information

Reporting Summary

## Data Availability

The raw data of Figs. 2, 3 and 5, 6, Supplementary Figs. 1–6, 8–15, 17, 19, 21, 22, and 24 can be found in the Source Data. Source data are provided with this paper. The authors declare that all other data supporting the findings of this study are within the article and its Supplementary Information file.

## References

[1] Ginzburg S (2017). Focal ablation therapy for renal cancer in the era of active surveillance and minimally invasive partial nephrectomy. Nat. Rev. Urol..

[2] Choi JW (2021). Radiofrequency ablation using a separable clustered electrode for the treatment of hepatocellular carcinomas: a randomized controlled trial of a dual-switching monopolar mode versus a single-switching monopolar mode. Korean J. Radiol..

[3] Kim Y-S (2013). Ten-year outcomes of percutaneous radiofrequency ablation as first-line therapy of early hepatocellular carcinoma: analysis of prognostic factors. J. Hepatol..

[4] Wahl DR (2016). Outcomes after stereotactic body radiotherapy or radiofrequency ablation for hepatocellular carcinoma. J. Clin. Oncol..

[5] Poulou LS (2015). Percutaneous microwave ablation vs radiofrequency ablation in the treatment of hepatocellular carcinoma. World J. Hepatol..

[6] De Baere T (2015). Radiofrequency ablation is a valid treatment option for lung metastases: experience in 566 patients with 1037 metastases. Ann. Oncol..

[7] Breen DJ (2015). Image-guided ablation of primary liver and renal tumours. Nat. Rev. Clin. Oncol..

[8] Lemdani K (2018). Therapeutic and cytotoxic responses after radiofrequency ablation combined to in situ immunomodulation and PD1 blockade in colorectal cancer. J. Clin. Oncol..

[9] Franco-Mahecha OL (2019). Effect of radiofrequency ablation (RFA) combined with anti-CTLA-4 and anti-PD1 in a preclinical melanoma model. J. Clin. Oncol..

[10] Waitz R (2012). Potent induction of tumor immunity by combining tumor cryoablation with anti–CTLA-4 therapy. Cancer Res..

[11] Shi L (2019). Inflammation induced by incomplete radiofrequency ablation accelerates tumor progression and hinders PD-1 immunotherapy. Nat. Commun..

[12] Dong Z (2019). Amplification of tumor oxidative stresses with liposomal fenton catalyst and glutathione inhibitor for enhanced cancer chemotherapy and radiotherapy. Nano Lett..

[13] Xue C-C (2020). Tumor microenvironment-activatable Fe-doxorubicin preloaded amorphous CaCO3 nanoformulation triggers ferroptosis in target tumor cells. Sci. Adv..

[14] Chen Q (2019). In situ sprayed bioresponsive immunotherapeutic gel for post-surgical cancer treatment. Nat. Nanotechnol..

[15] Tang Z (2019). Chemodynamic therapy: tumour microenvironment-mediated fenton and fenton-like reactions. Angew. Chem. Int. Ed..

[16] Wang B (2019). Sequential intercellular delivery nanosystem for enhancing ROS-induced antitumor therapy. Nano Lett..

[17] Lee J-Y (2020). Polyunsaturated fatty acid biosynthesis pathway determines ferroptosis sensitivity in gastric cancer. Proc. Natl Acad. Sci. USA.

[18] Ursini F (2020). Lipid peroxidation and ferroptosis: the role of GSH and GPx4. Free Radic. Biol. Med..

[19] Jiang X (2021). Ferroptosis: mechanisms, biology and role in disease. Nat. Rev. Mol. Cell Biol..

[20] Zou Y (2020). Cytochrome P450 oxidoreductase contributes to phospholipid peroxidation in ferroptosis. Nat. Chem. Biol..

[21] Zhou Z (2017). Activatable singlet oxygen generation from lipid hydroperoxide nanoparticles for cancer therapy. Angew. Chem. Int. Ed..

[22] Shintoku R (2017). Lipoxygenase-mediated generation of lipid peroxides enhances ferroptosis induced by erastin and RSL3. Cancer Sci..

[23] Liu F (2020). Suppression of membranous LRP5 recycling, wnt/beta-catenin signaling, and colon carcinogenesis by 15-lox-1 peroxidation of linoleic acid in PI3P. Cell Rep..

[24] Rothe T (2015). 12/15-lipoxygenase–mediated enzymatic lipid oxidation regulates DC maturation and function. J. Clin. Invest..

[25] Yang WS (2016). Ferrootosis: death by lipid peroxidation. Trends Cell Biol..

[26] Zhang B (2019). Polymer dots compartmentalized in liposomes as a photocatalyst for in situ hydrogen therapy. Angew. Chem. Int. Ed..

[27] Stockwell BR (2017). Ferroptosis: a regulated cell death nexus linking metabolism, redox biology, and disease. Cell.

[28] Kagan VE (2017). Oxidized arachidonic and adrenic PEs navigate cells to ferroptosis. Nat. Chem. Biol..

[29] Levental KR (2017). ω-3 polyunsaturated fatty acids direct differentiation of the membrane phenotype in mesenchymal stem cells to potentiate osteogenesis. Sci. Adv..

[30] Balabushevich NG (2019). Hybrid CaCO3-mucin crystals: effective approach for loading and controlled release of cationic drugs. Mater. Des..

[31] Zhu Y (2020). CaCO3-assisted preparation of pH-responsive immune-modulating nanoparticles for augmented chemo-immunotherapy. Nano Micro Lett..

[32] Wang S (2018). Exploration of antigen Induced CaCO3 nanoparticles for therapeutic vaccine. Small.

[33] Artzi N (2009). Aldehyde-amine chemistry enables modulated biosealants with tissue-specific adhesion. Adv. Mater..

[34] Dong Z (2020). Synthesis of CaCO3-based nanomedicine for enhanced sonodynamic therapy via amplification of tumor oxidative stress. Chem.

[35] Hassannia B (2019). Targeting ferroptosis to iron out cancer. Cancer Cell.

[36] Wan C (2020). Irradiated tumor cell-derived microparticles mediate tumor eradication via cell killing and immune reprogramming. Sci. Adv..

[37] Yu B (2020). Magnetic field boosted ferroptosis-like cell death and responsive MRI using hybrid vesicles for cancer immunotherapy. Nat. Commun..

[38] Ruiz-de-Angulo A (2020). Chemically programmed vaccines: iron catalysis in nanoparticles enhances combination immunotherapy and immunotherapy-promoted tumor ferroptosis. iScience.

[39] Zhang J (2021). Heparanase-driven sequential released nanoparticles for ferroptosis and tumor microenvironment modulations synergism in breast cancer therapy. Biomaterials.

[40] Mukerabigwi JF (2019). Polymersome nanoreactors with tumor pH-triggered selective membrane permeability for prodrug delivery, activation, and combined oxidation-chemotherapy. J. Control Release.

[41] Galluzzi L (2020). Consensus guidelines for the definition, detection and interpretation of immunogenic cell death. J. Immunother. Cancer.

[42] Zhou F (2019). Tumor microenvironment-activatable prodrug vesicles for nanoenabled cancer chemoimmunotherapy combining immunogenic cell death induction and CD47 blockade. Adv. Mater..

[43] Chao Y (2020). Localized cocktail chemoimmunotherapy after in situ gelation to trigger robust systemic antitumor immune responses. Sci. Adv..

[44] Phuengkham H (2019). Nanoengineered immune niches for reprogramming the immunosuppressive tumor microenvironment and enhancing cancer immunotherapy. Adv. Mater..

[45] Doerdelmann G (2014). Calcium phosphate increases the encapsulation efficiency of hydrophilic drugs (proteins, nucleic acids) into poly(D,L-lactide-co-glycolide acid) nanoparticles for intracellular delivery. J. Mater. Chem. B.

[46] Grossman S (1979). Determination of the activity of lipoxygenase (lipoxidase). Methods Biochem. Anal..

[47] Ngin P (2021). Immobilization of soybean lipoxygenase on nanoporous rice husk silica by adsorption: Retention of enzyme function and catalytic potential. Molecules.

[48] Meng Z (2019). Light-triggered in situ gelation to enable robust photodynamic-ommunotherapy by repeated stimulations. Adv. Mater..

