# Supplementary material for: Tumor-killing nanoreactors fueled by tumor debris can enhance radiofrequency ablation therapy and boost antitumor immune responses
Source: Nat Commun. 2021 Jul 14;12:4299. doi: 10.1038/s41467-021-24604-9 (PMC8280226; doi:10.1038/s41467-021-24604-9)
Supplement: Supplementary file 1 — Supplementary information [file 41467_2021_24604_MOESM1_ESM.pdf]

Supplementary Information

Tumor-killing Nanoreactors Fueled by Tumor Debris Can Enhance Radiofrequency Ablation Therapy and Boost Antitumor Immune Responses

Running Title: Ablating a Tumor using Its Debris as the Fuel

Zhijuan Yang[1], Yujie Zhu[1], Ziliang Dong[1], Wei Li[1], Nailin Yang[1], Xianwen Wang[1], Liangzhu Feng[1]*, Zhuang Liu[1,2] *

1, Jiangsu Key Laboratory for Carbon-Based Functional Materials & Devices, Institute of Functional Nano & Soft Materials (FUNSOM), Soochow University, 199 Ren'ai Road, Suzhou, 215123, Jiangsu, PR China
2, Macao Institute of Materials Science and Engineering, Macau University of Science and Technology, Taipa, 999078 Macau SAR, China

Email: zliu@suda.edu.cn, lzfeng@suda.edu.cn

Supplementary Figures:

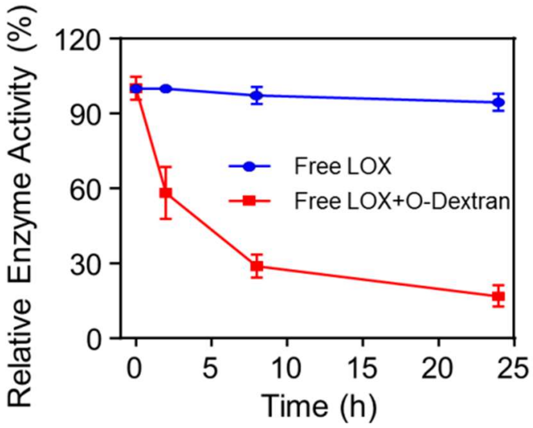

Supplementary Fig. 1 Time-dependent relative enzyme activity of free LOX (0.5 mg mL^{-1}) in the presence and absence of oxidized dextran (o-dextran, 2 mg mL^{-1}), n = 3 biologically independent samples, data was represented as mean ± SD.

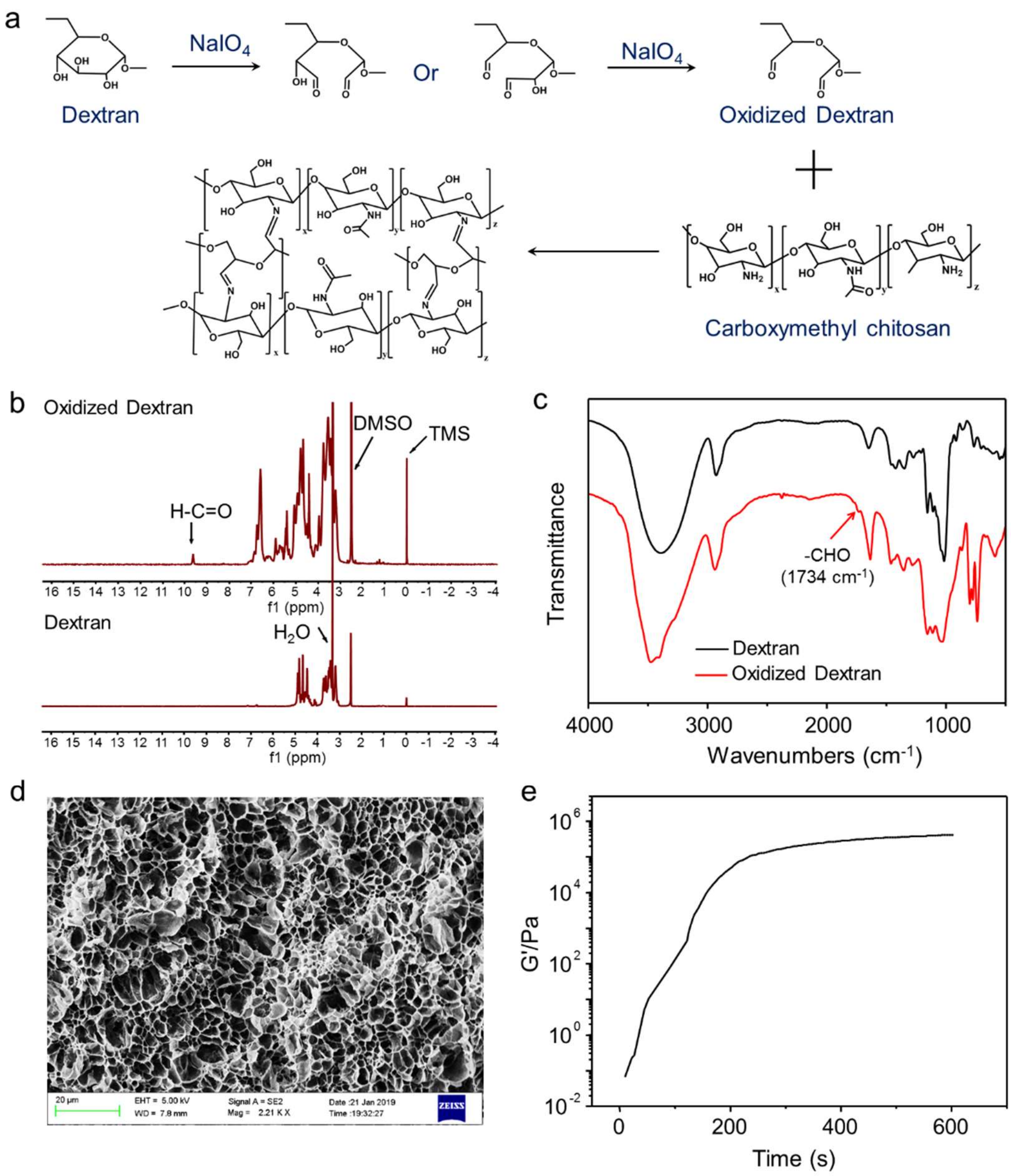

Supplementary Fig. 2 Synthesis and characterization of adhesive glue. (a) Schematic illustration of the synthesis process of oxidative dextran, and the chemical formation process of such oxidized dextran with commercial carboxymethyl chitosan. (b&c) The proton nuclear magnetism resonance (^{1}H NMR, b) and FT-IR (c) spectra of commercial dextran and as-prepared oxidized dextran. (d&e) SEM images (d) and the time-dependent storage modulus (G', e) of the adhesive glue formed with oxidized dextran and carboxymethyl chitosan. A representative SEM image of three independent experiments is shown in Figure d.

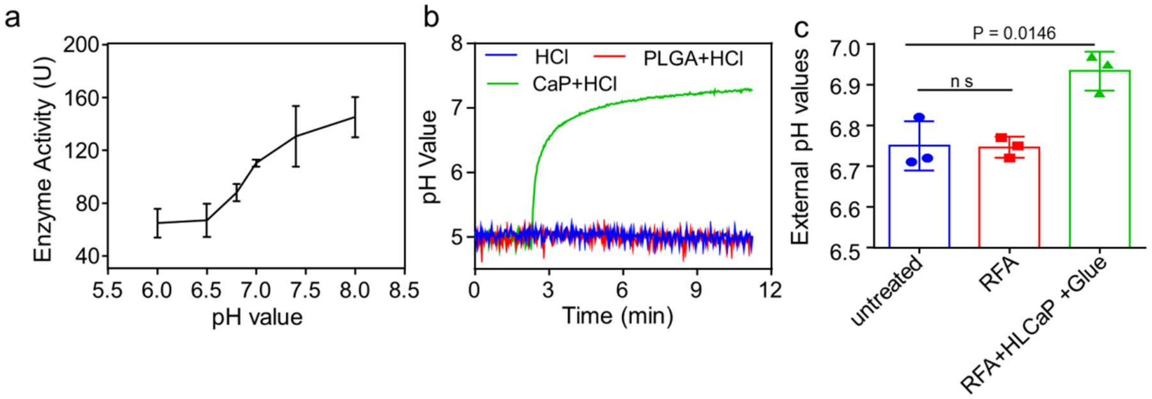

Supplementary Fig. 3 (a) The catalytic capacity of free LOX molecules determined at varying pH values (n = 3 biologically independent samples). (b) The capacity of CaP NPs in neutralizing protons in a dilute HCl solution at pH 5.0. (c) External pH values of intact untreated tumors, residual tumor mass of these tumors with RFA treatment alone and RFA treatment plus HLCaP NRs /glue injection recorded using an invasive microelectrode (n = 3 biologically independent animals). Data in Figure a&c were represented as mean ± SD.

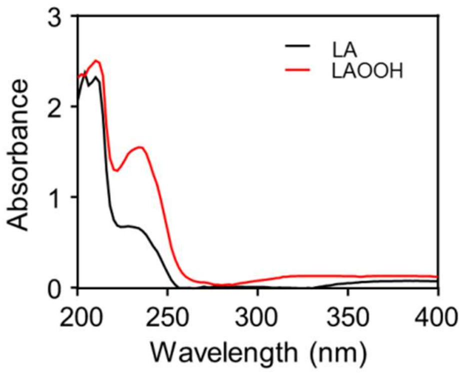

Supplementary Fig. 4. UV-vis spectra of native LA (0.9 μg mL^{-1}) and corresponding LAOOH prepared by incubating commercial LA (0.9 μg mL^{-1}) with LOX at 37 ºC for 3 min.

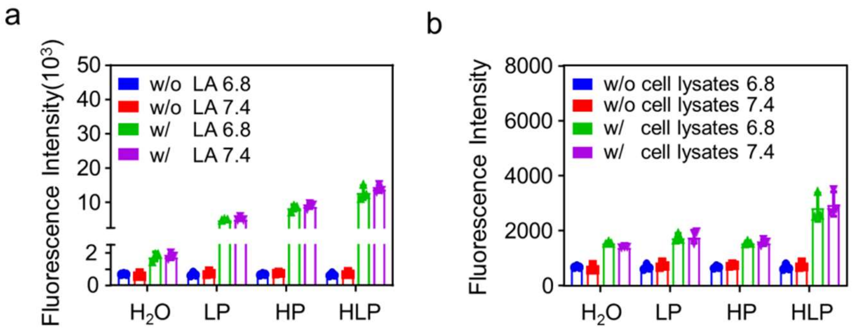

Supplementary Fig. 5 Lipid peroxidation generation capacities of LP, HP and HLP NPs quantified by incubating them with LA (a) or cell lysates (b) at pH 6.8 and 7.4 for 4 h, respectively. Data were represented as mean ± SD, n = 3 biologically independent samples.

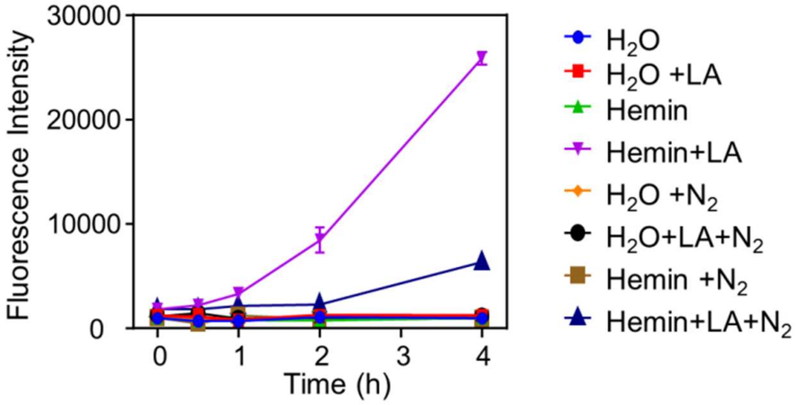

Supplementary Fig. 6. The capacities of hemin in promoting the propagation of lipid peroxidation from linoleic acids (LA) in the presence of oxygen and nitrogen. It was shown the hemin in the presence of oxygen could effectively promote the propagation of LA from these auto-oxidized LA, which is generated via the dissolved oxygen mediated oxidation of LA. Data was represented as mean ± SD, n = 3 biologically independent samples.

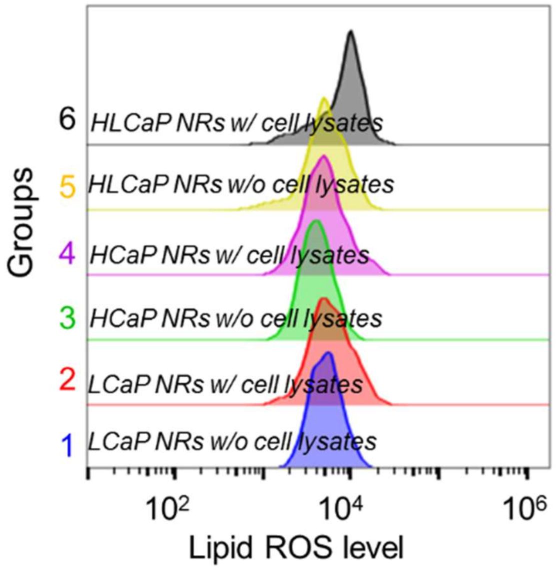

Supplementary Fig. 7 Flow cytometric analysis of 4T1 cells incubated with HCaP NPs, LCaP NPs and HLCaP NRs in the presence (w/) or absence (w/o) of cancer cell lysates for 6 h, followed by being stained with lipid peroxides probe of BODIPY-C11.

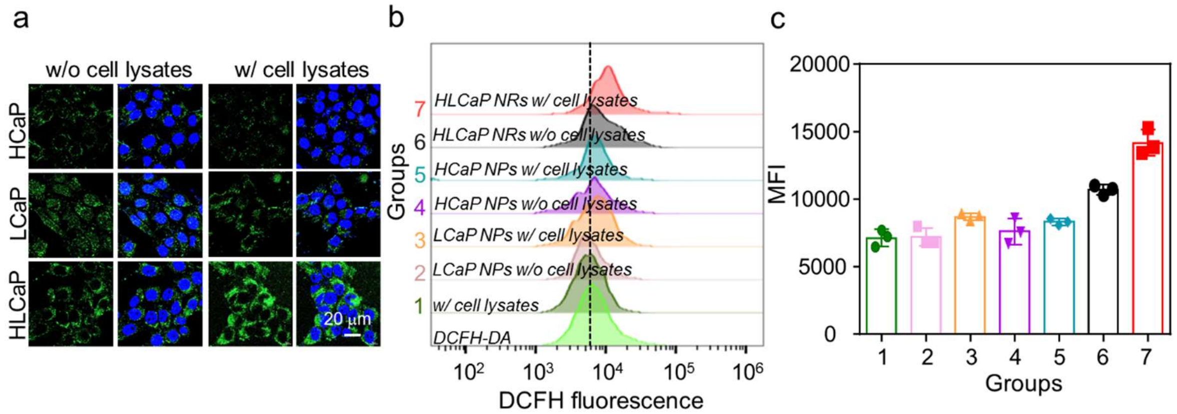

Supplementary Fig. 8 (a&b) Representative confocal images (a) and flow cytometric analysis (b) of intracellular ROS generation of 4T1 cells incubated with LCaP NPs, HCaP NPs and HLCaP NRs in the presence or absence of cancer cell lysates for 4 h, respectively, followed by stained with DCFH-DA. (c) Semi-quantitative analysis of mean fluorescence intensity (MFI) of these treated cells based on Figure b. Data were represented as mean ± SD, n = 3 biologically independent samples. A representative image of three biologically independent samples from each group is shown in Figure a.

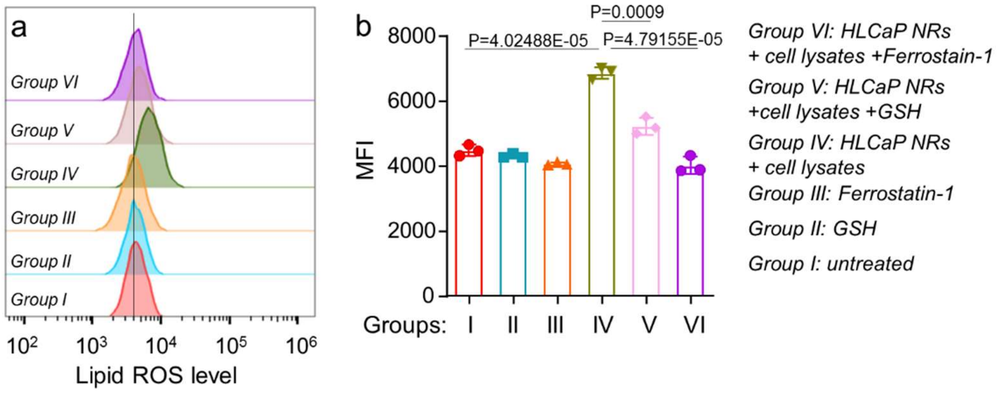

Supplementary Fig. 9 Flow cytometric analysis of intracellular lipid peroxidation (a) and corresponding mean fluorescence intensity (b) of 4T1 cells post various treatments as indicated (n = 3 biologically independent samples), data in Figure b was represented as mean ± SD. P values calculated by two-tailed student's t-test were shown on figure.

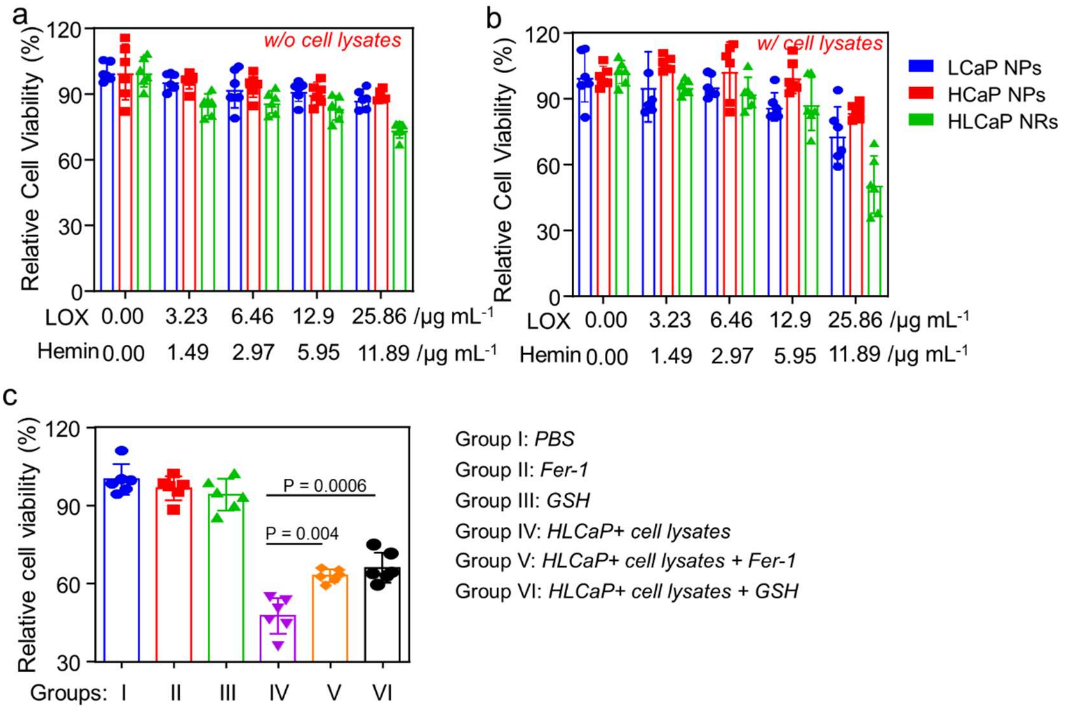

Supplementary Fig. 10 Relative viabilities of HepG2 cells incubated with HCaP NPs, LCaP NPs and HLCaP NRs in the absence (a) or presence (b) of cancer cell lysates for 24 h before being determined by MTT assay. (c) Relative cell viabilities of HepG2 cells post various treatments as indicated. Data were represented as mean ± SD, n = 6 biologically independent samples, P values calculated by two-tailed student's t-test were shown on figure.

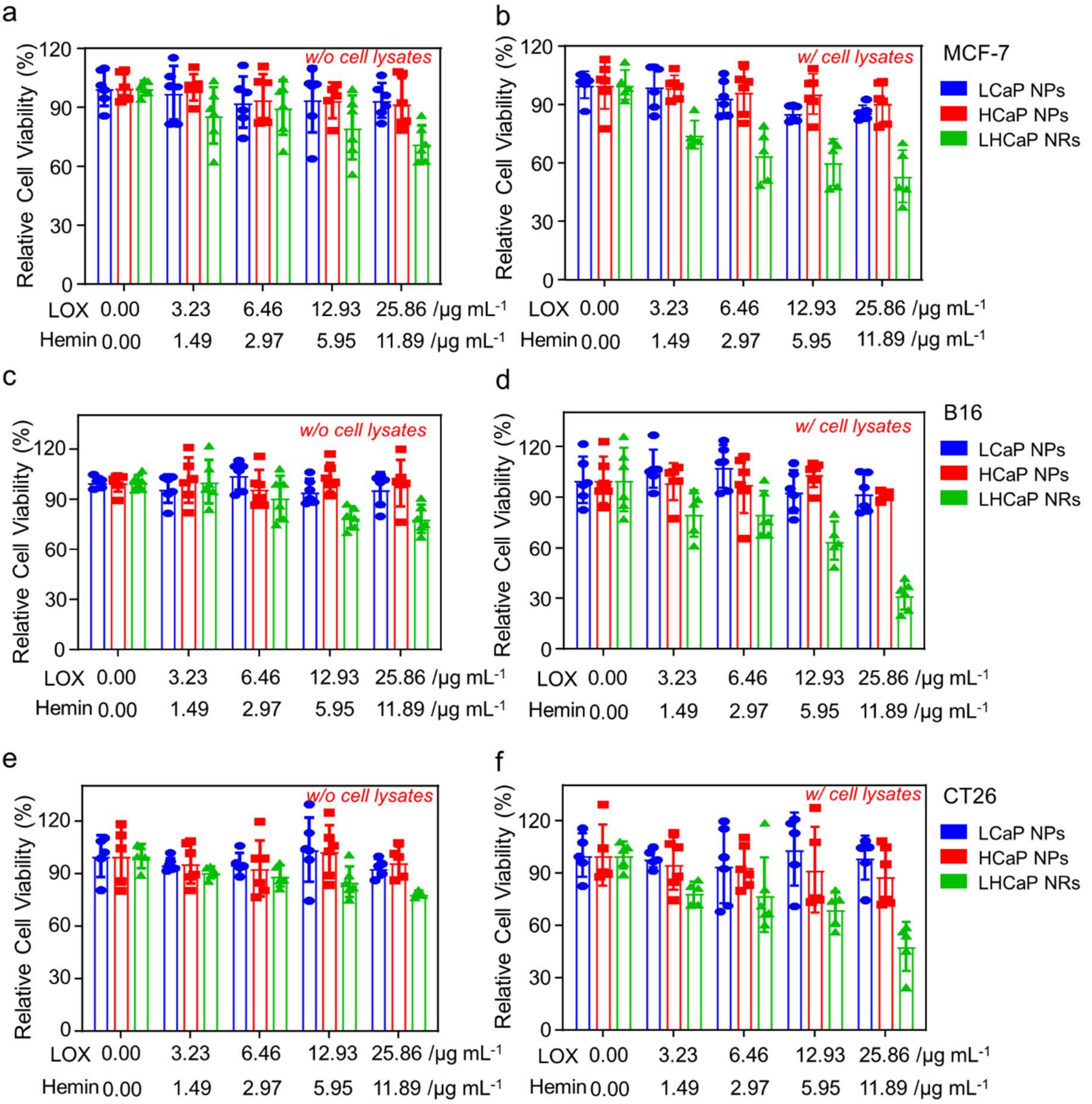

Supplementary Fig. 11 *In vitro* therapeutic efficacay of HLCaP NRs against MCF-7 cells (a&b), B16 cells (c&d), and CT26 cells (e&f) treated in the presence or absence of cancer cell lysates, respectively. Data were represented as mean ± SD, n = 6 biologically independent samples.

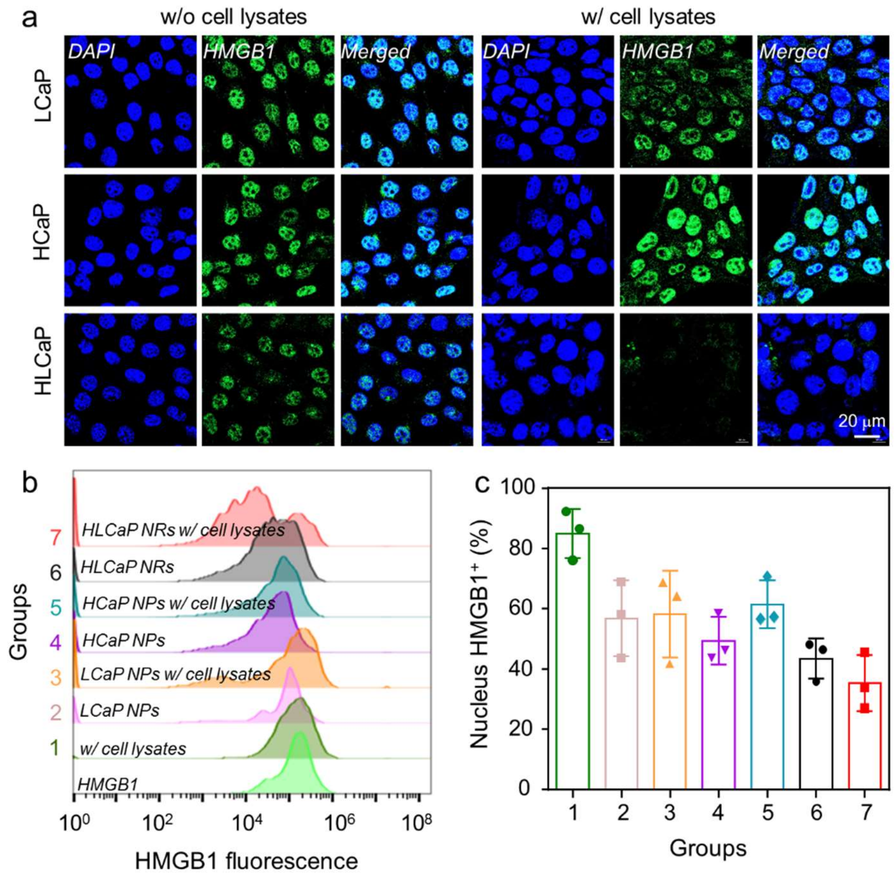

Supplementary Fig. 12 Representative confocal imaging (a) and flow cytometric analysis (b) of HMGB1 release of 4T1 cells incubated with LCaP NPs, HCaP NPs and HLCaP NRs in the presence or absence of cell lysates for 24 h, respectively, followed by anti-HMGB1 staining. (c) Semi-quantitative analysis of the percentage of HMGB1 positive cells based on Figure b, data were represented as mean ± SD, n = 3 biologically independent samples. A representative image of three biologically independent samples from each group is shown in Figure a.

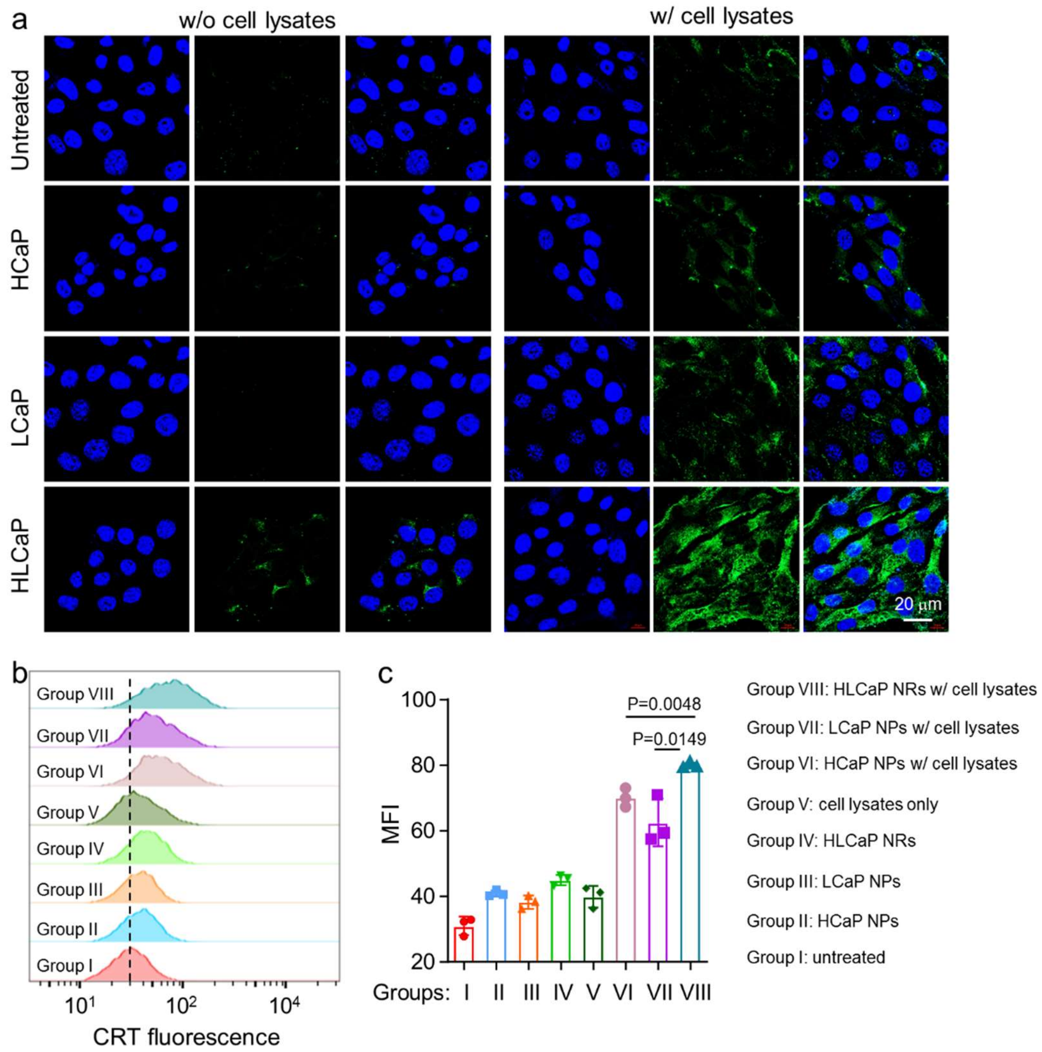

Supplementary Fig. 13 Representative confocal imaging (a) and flow cytometric analysis (b) of CRT expression on 4T1 cells incubated with LCaP NPs, HCaP NPs and HLCaP NRs in the presence or absence of cell lysates for 8 h as indicated, respectively, followed by anti-CRT staining. (c) Semi-quantitative analysis of CRT fluorescence intensity based on Figure b, data were represented as mean ± SD, n = 3 biologically independent samples. A representative image of three biologically independent samples from each group is shown in Figure a.

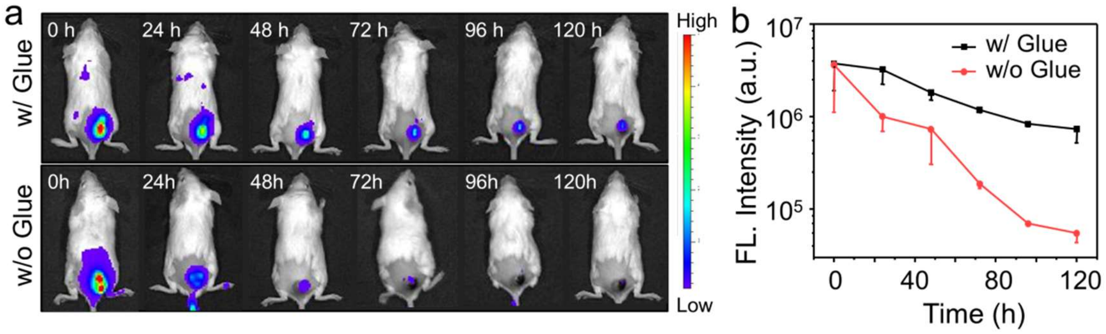

Supplementary Fig. 14 (a) Representative *in vivo* fluorescence imaging of 4T1 tumor-bearing mice with intratumoral injection of Cy5.5 labeled HLCaP NRs in the presence or absence of adhesive glue at indicated time points post RFA treatment. (b) Semi-quantitative analysis of remained Cy5.5 fluorescence in tumor regions based on the fluorescence images shown in Figure a, data were represented as mean ± SD, n = 3 biologically independent samples.

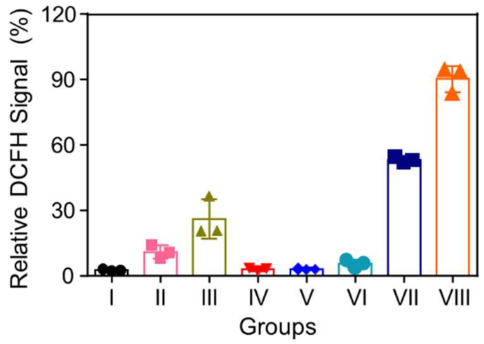

Supplementary Fig. 15 Semi-quantitative analysis of DCFH fluorescence intensities of the tumor slices as shown in Figure 3e, data were represented as mean ± SD, n = 3 biologically independent samples,.

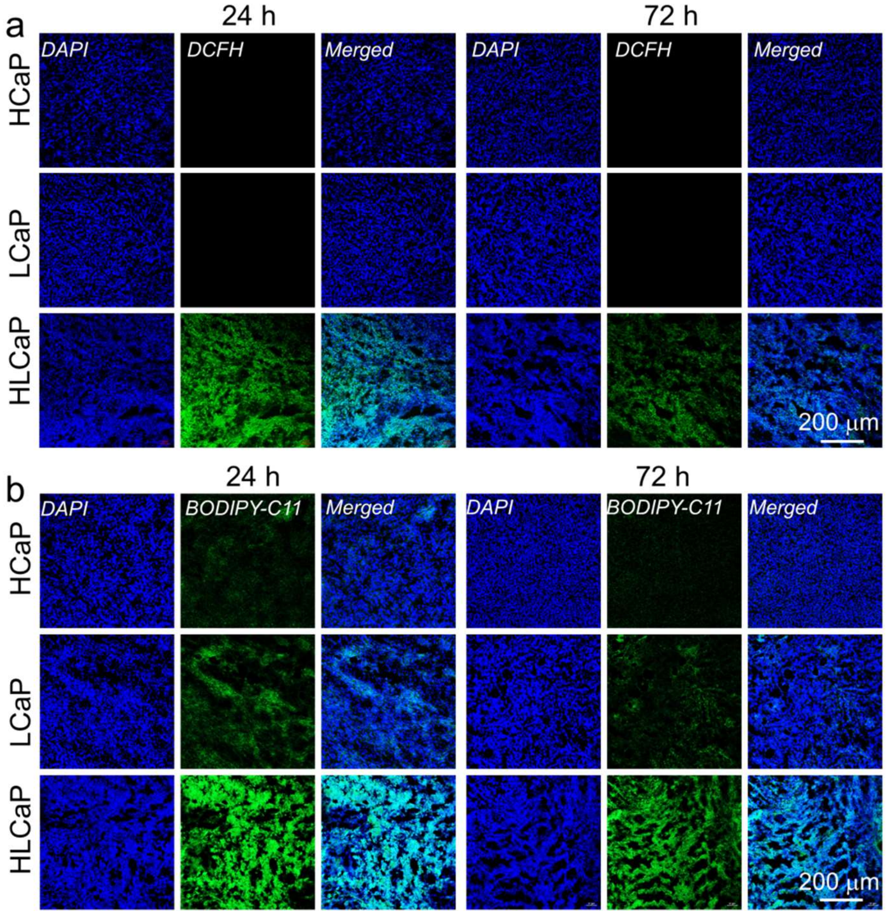

Supplementary Fig. 16 Confocal images of tumor slices collected from 4T1 tumor-bearing mice at 24 h and 72 h post different treatments as indicated, and subsequently stained with DCFH-DA (a) and BODIPY^{TM} 581/591 C11 (b), respectively. A representative image of three biologically independent replicates from each group is shown in Figure a-b.

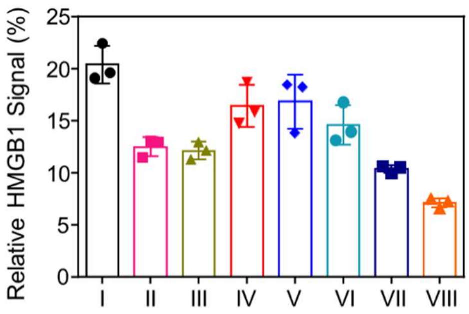

Supplementary Fig. 17 Semi-quantitative analysis of HMGB1 signals of the tumor slices as shown in figure 3f, data was represented as mean ± SD, n = 3 biologically independent samples.

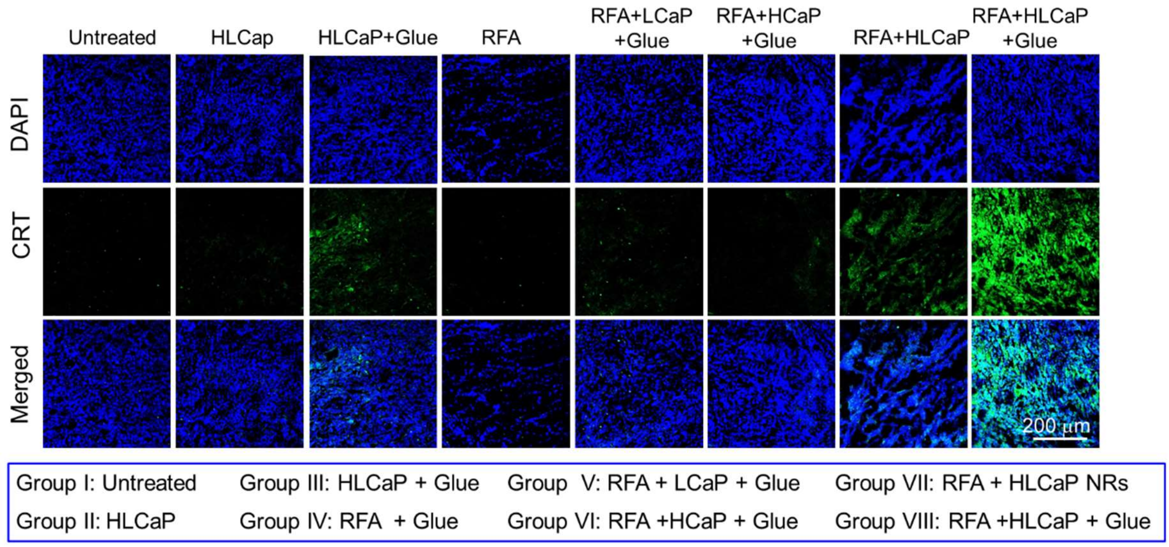

Supplementary Fig. 18 Confocal images of tumor slices collected from 4T1 tumor-bearing mice after different treatments as indicated for 24 h and stained with CRT primary antibodies and corresponding Alexa 488 conjugated secondary antibodies. A representative image of three biologically independent samples from each group is shown.

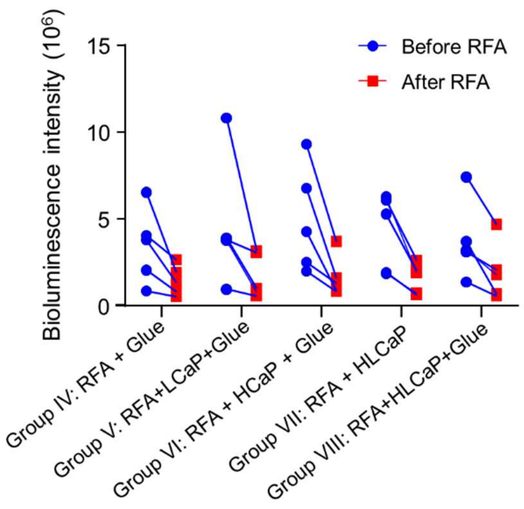

Supplementary Fig. 19 Semi-quantitative analysis of bioluminescence intensity of Luc-4T1 tumors before and after RFA treatment (50 W, 2 min) based on the *in vivo* bioluminescence imaging shown in Figure 4b.

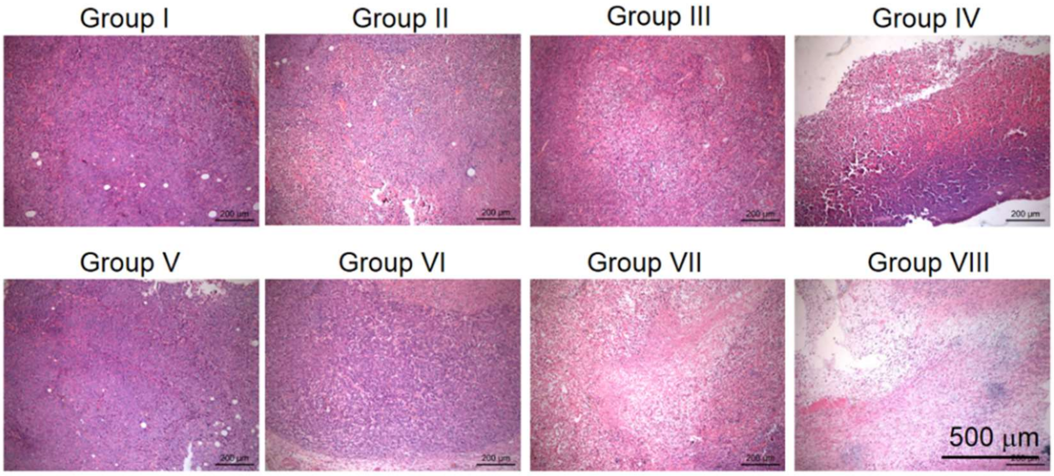

Supplementary Fig. 20 The micrographs of H&E-stained tumor slices collected at 24 h from 4T1 tumor-bearing mice after different treatments. A representative image of three biologically independent samples from each group is shown.

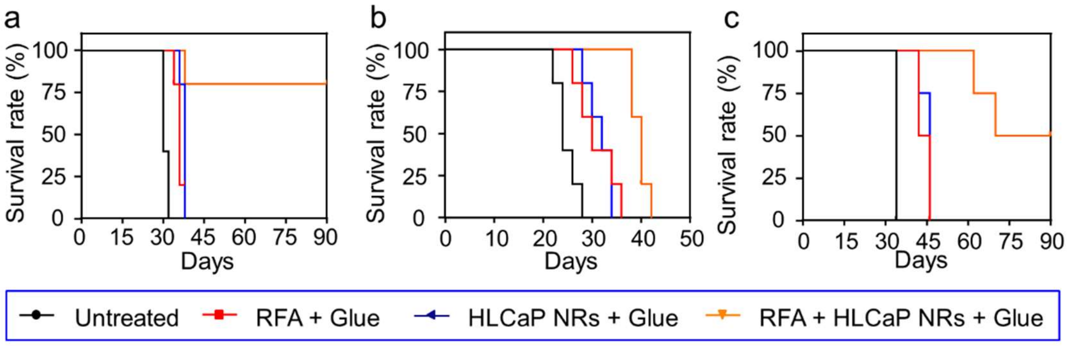

Supplementary Fig. 21 Mobility free survival rates of animals bearing H22 tumors (a, n = 5), PDX tumors (b, n = 5) and VX2 tumors (c, n = 4) post different treatments as indicated. The mice and rabbits were euthanized when their tumor volumes were larger than 1000 and 5000 mm^3, respectively.

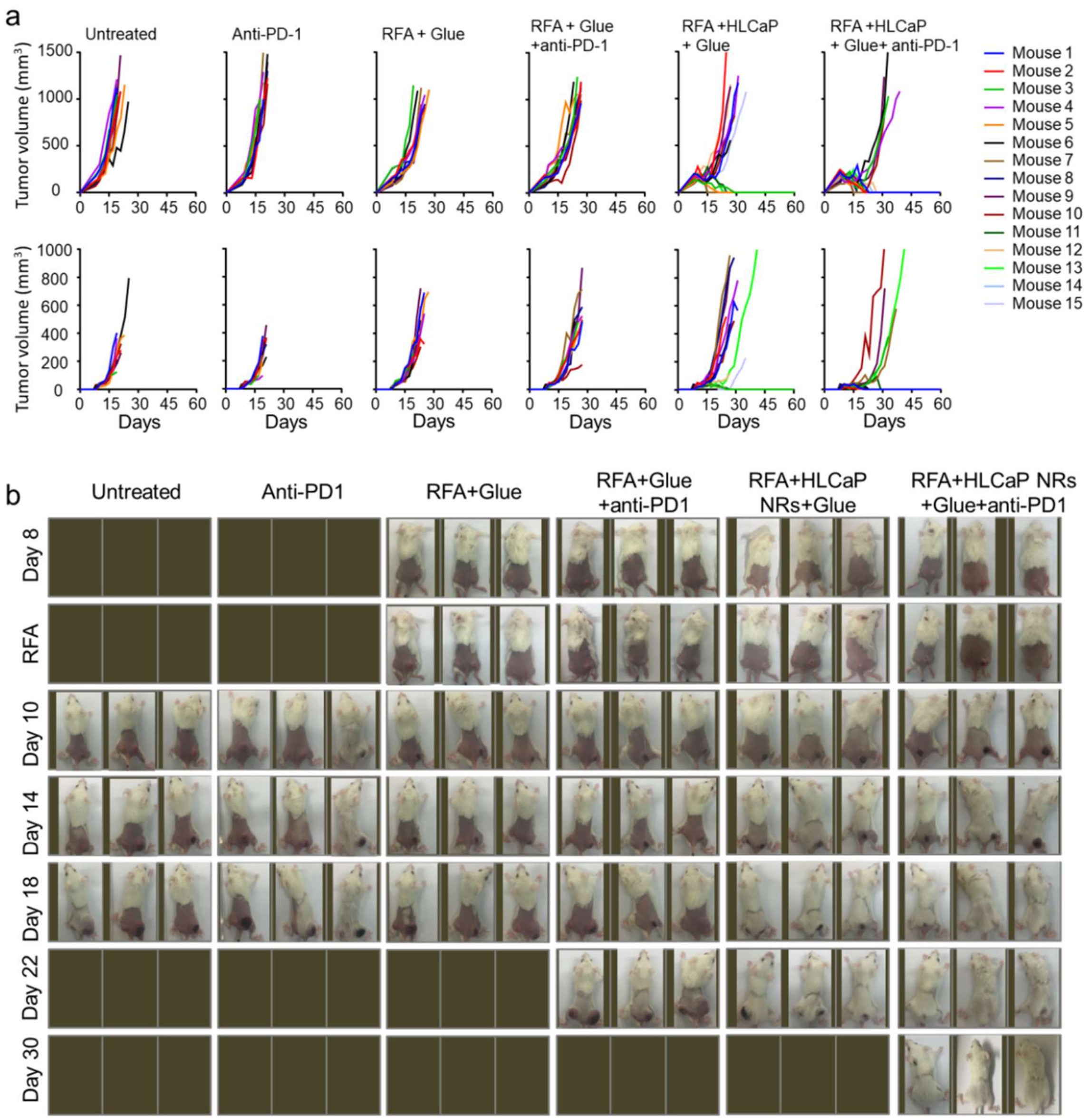

Supplementary Fig. 22 (a) Individual tumor growth curves of both primary treated 4T1 tumors (up panel) and distant untreated tumors (down panel) of each group of mice with different treatments as indicated (n = 10 or 15). (b) Representative photographs of 3 mice randomly picked out from each group recorded at varying time intervals as indicated.

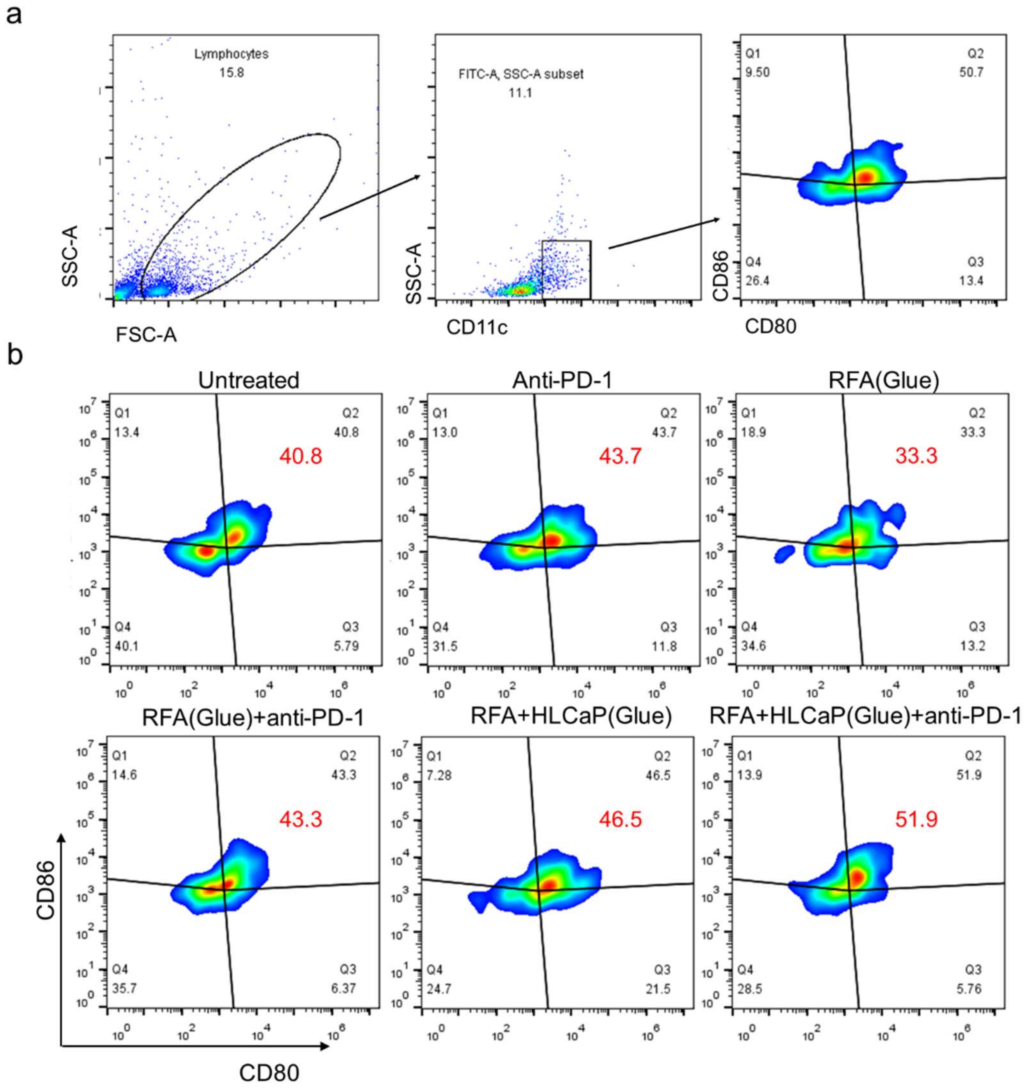

Supplementary Fig. 23 (a) Gating strategy to determine the percentage of $CD11c^{+}CD80^{+}CD86^{+}$ DC cells. (b) Representative flow cytometric analysis of the DC maturation in the drain lymph nodes adjacent to the primary tumors based on 5 biologically independent samples.

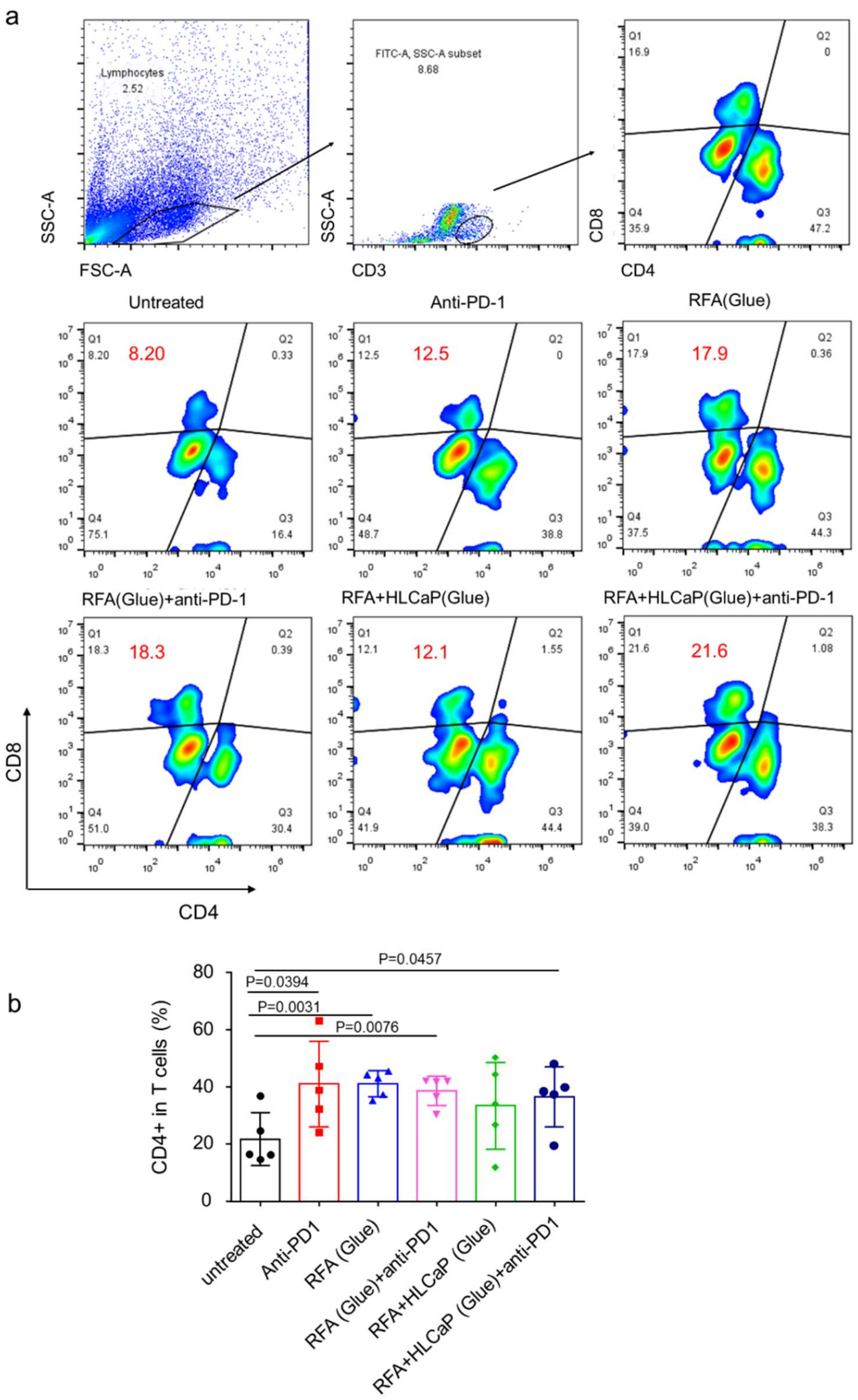

Supplementary Fig. 24 (a) Gating strategy to determine the percentages of $CD3^+CD8^+$ T cells and $CD3^+CD4^+$ T cells, respectively (up panel). Representative flow cytometric analysis of the frequency of $CD3^+CD8^+$ T cells in the distant tumors based on 5 biologically independent samples. (b) The frequencies of $CD3^+CD4^+$ T cells inside the distant tumors post various treatments as indicated, data was represented as mean ± SD, n = 5 biologically independent animals.

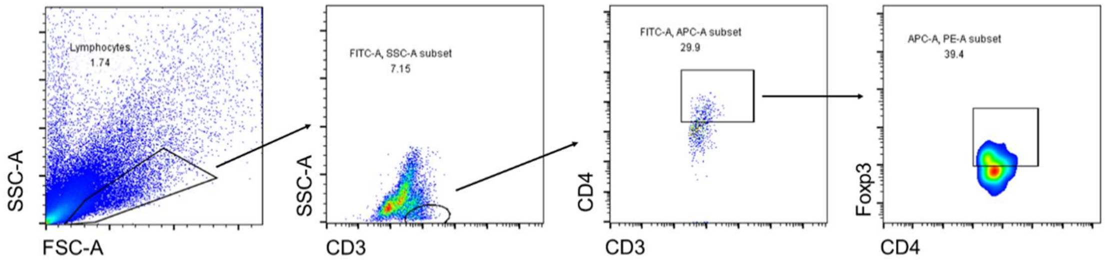

Supplementary Fig.25 Gating strategy to determine the percentage of Tregs in the distant tumors of mice post various treatments as shown in Figure 6.
