# Supplementary material for: Tumor-killing nanoreactors fueled by tumor debris can enhance radiofrequency ablation therapy and boost antitumor immune responses
Source: Nat Commun. 2021 Jul 14;12:4299. doi: 10.1038/s41467-021-24604-9 (PMC8280226; doi:10.1038/s41467-021-24604-9)
Supplement: Supplementary file 2 — Reporting Summary [file 41467_2021_24604_MOESM2_ESM.pdf]

nature research

Corresponding author(s): Zhuang liu

Last updated by author(s): Jun 15, 2021

Reporting Summary

Nature Research wishes to improve the reproducibility of the work that we publish. This form provides structure for consistency and transparency in reporting. For further information on Nature Research policies, see our Editorial Policies and the Editorial Policy Checklist.

Statistics

For all statistical analyses, confirm that the following items are present in the figure legend, table legend, main text, or Methods section.

n/a	Confirmed	
☐	☒	The exact sample size (*n*) for each experimental group/condition, given as a discrete number and unit of measurement
☐	☒	A statement on whether measurements were taken from distinct samples or whether the same sample was measured repeatedly
☐	☒	The statistical test(s) used AND whether they are one- or two-sided<br>*Only common tests should be described solely by name; describe more complex techniques in the Methods section.*
☐	☒	A description of all covariates tested
☐	☒	A description of any assumptions or corrections, such as tests of normality and adjustment for multiple comparisons
☐	☒	A full description of the statistical parameters including central tendency (e.g. means) or other basic estimates (e.g. regression coefficient) AND variation (e.g. standard deviation) or associated estimates of uncertainty (e.g. confidence intervals)
☐	☒	For null hypothesis testing, the test statistic (e.g. *F*, *t*, *r*) with confidence intervals, effect sizes, degrees of freedom and *P* value noted<br>*Give P values as exact values whenever suitable.*
☒	☐	For Bayesian analysis, information on the choice of priors and Markov chain Monte Carlo settings
☒	☐	For hierarchical and complex designs, identification of the appropriate level for tests and full reporting of outcomes
☐	☒	Estimates of effect sizes (e.g. Cohen's *d*, Pearson's *r*), indicating how they were calculated

Our web collection on statistics for biologists contains articles on many of the points above.

Software and code

Policy information about availability of computer code

Data collection	No software was used for date collection
Data analysis	All statistical analyses were performed on Graphpad Prism 6, Excel 2013, Origin 9.1. Living Image software (Perkin Elmer) was used to analyse bioluminescent images. All the flow cytometry data were processed using FlowJo.

For manuscripts utilizing custom algorithms or software that are central to the research but not yet described in published literature, software must be made available to editors and reviewers. We strongly encourage code deposition in a community repository (e.g. GitHub). See the Nature Research guidelines for submitting code & software for further information.

Data

Policy information about availability of data

All manuscripts must include a data availability statement. This statement should provide the following information, where applicable:

- Accession codes, unique identifiers, or web links for publicly available datasets
- A list of figures that have associated raw data
- A description of any restrictions on data availability

All the data supporting the findings of this study are available within the article and its Supplementary Information file and from the corresponding authors upon reasonable request. Source data are provided with this paper.

Field-specific reporting

Please select the one below that is the best fit for your research. If you are not sure, read the appropriate sections before making your selection.

- [x] Life sciences
- [ ] Behavioural & social sciences
- [ ] Ecological, evolutionary & environmental sciences

For a reference copy of the document with all sections, see nature.com/documents/nr-reporting-summary-flat.pdf

Life sciences study design

All studies must disclose on these points even when the disclosure is negative.

Sample size	No statistical methods were used to predetermine the sample sizes. It is impossible to predict the magnitude of experimental variation between animals based on our current knowledge. The group sizes (at least three animals per treatment group) represents the minimum number animals needed to reach statistical significance ($p < 0.05$) between experimental groups.
Data exclusions	No data were excluded.
Replication	Experiments were repeated and experimental findings were reproducible.
Randomization	Mice and rabbits were randomly allocated into experimental groups.
Blinding	No blinding was done in this study. Most of the studies contained multiple steps (including the material preparation, tumor treatment, and so on) and the scientists must keep careful track of conditions. It would be exceedingly difficult to blind such studies.

Reporting for specific materials, systems and methods

We require information from authors about some types of materials, experimental systems and methods used in many studies. Here, indicate whether each material, system or method listed is relevant to your study. If you are not sure if a list item applies to your research, read the appropriate section before selecting a response.

Materials & experimental systems

n/a	Involved in the study	
[ ]	[x]	Antibodies
[ ]	[x]	Eukaryotic cell lines
[x]	[ ]	Palaeontology and archaeology
[ ]	[x]	Animals and other organisms
[x]	[ ]	Human research participants
[x]	[ ]	Clinical data
[x]	[ ]	Dual use research of concern

Methods

n/a	Involved in the study	
[x]	[ ]	ChIP-seq
[ ]	[x]	Flow cytometry
[x]	[ ]	MRI-based neuroimaging

Antibodies

Antibodies used	anti-CD3-FITC (Biolegend, clone 17A2, Catalog: 100204), anti-CD4-APC (Biolegend, clone GK1.5, Catalog: 100412), anti-CD8-PE (Biolegend, clone 53-6.7, Catalog: 100708), and anti-Foxp3-PE (Biolegend, clone MF-14, Catalog: 126404), anti-CD11c-FITC(Biolegend, clone N418, Catalog: 117306), anti-CD80-PE (Biolegend, clone 16-10A1, Catalog: 104708), anti-CD86-APC (Biolegend, clone GL-1, Catalog: 105012), Anti-HMGB1 antibody (MultiSciences, 70-ab40050-100), Anti-CRT antibody (Abcam, ab2907), Alexa 488-conjugated secondary antibody (Jackson,111-545-003), Anti-PD-1(Bioxcell,BE0146)

Validation	All antibodies were verified by the supplier and each lot has been quality tested. All validation statements can be found on the respective antibody website:<br>1. Anti-mouse-CD8a-PE: https://www.biolegend.com/en-us/products/pe-anti-mouse-cd8a-antibody-155<br>2. Anti-mouse-CD3-FITC: https://www.biolegend.com/en-us/products/fitc-anti-mouse-cd3-antibody-45<br>3. Anti-mouse-CD4-APC: https://www.biolegend.com/en-us/products/apc-anti-mouse-cd4-antibody-245<br>4. Anti-mouse-Foxp3-PE: https://www.biolegend.com/en-us/products/pe-anti-mouse-foxp3-antibody-4660<br>5. Anti-mouse-CD11c-FITC:https://www.biolegend.com/en-us/products/fitc-anti-mouse-cd11c-antibody-1815<br>6. Anti-mouse-CD80-PE:https://www.biolegend.com/en-us/products/pe-anti-mouse-cd80-antibody-43<br>7. Anti-mouse-CD86-APC:https://www.biolegend.com/en-us/products/apc-anti-mouse-cd86-antibody-2896<br>8. Anti-HMGB1 antibody:http://42.244.43.54/Search/Products/Detail?id=00e5e8b4-619d-441f-af93-cb43bacc3482<br>9. Anti-CRT antibody:https://www.abcam.cn/calreticulin-antibody-er-marker-ab2907.html<br>10. Alexa 488-conjugated secondary antibody:https://www.jacksonimmuno.com/catalog/products/111-545-003<br>11.Anti-PD-1:https://bxcell.com/product/invivomab-anti-m-pd-1/

Eukaryotic cell lines

Policy information about cell lines

Cell line source(s)	Murine 4T1 breast cancer cells (SCSP-5056), murine CT26 colon cancer cells (TCM37), murine B16 melanoma cells, human HepG2 hepatocellular carcinoma cells (SCSP-510), murine B16 melanoma cells (TCM 2) and human MCF-7 breast cancer cells (SCSP-531) were obtained from the Cell Bank, Shanghai Institutes for Biological Sciences, Chinese Academy of Sciences. Luciferase-transfected 4T1 cells (Luc-4T1) was obtained from PerkinElmer Co as a gift, murine H22 hepatocellular carcinoma cells (ZQ0109) was obtained from Shanghai Zhongqiao Xinzhou Biological Technology Co., Ltd. Rabbit VX2 liver cancer cells (MZ-0769) was obtained from Ningbo Mingzhou Biological Technology Co., Ltd.
Authentication	Identity of the cell lines were frequently checked by their morphological features but have not been authenticated by the short tandem repeat (STR) profiling.
Mycoplasma contamination	All cell lines were tested for mycoplasma contamination. No mycoplasma contamination was found.
Commonly misidentified lines<br>(See ICLAC register)	No commonly misidentified cell lines are used in this study.

Animals and other organisms

Policy information about studies involving animals; ARRIVE guidelines recommended for reporting animal research

Laboratory animals	Six-week-old Balb/c female mice (18 ± 2g), Six-week-old female Balb/c nude mice (18 ± 2g) and two-month-old SPF new Zealand white rabbits (1 ± 0.5kg) were purchased from Laboratory Animal Center of Soochow University
Wild animals	The study did not involve wild animals.
Field-collected samples	The study did not involve samples collected from the field.
Ethics oversight	The animal experimental protocols were approved by the Ethics Committee of the Animal Experiment Center of Soochow University (Suzhou, China).

Note that full information on the approval of the study protocol must also be provided in the manuscript.

Flow Cytometry

Plots

Confirm that:

- [ ] The axis labels state the marker and fluorochrome used (e.g. CD4-FITC).
- [x] The axis scales are clearly visible. Include numbers along axes only for bottom left plot of group (a 'group' is an analysis of identical markers).
- [x] All plots are contour plots with outliers or pseudocolor plots.
- [x] A numerical value for number of cells or percentage (with statistics) is provided.

Methodology

Sample preparation	The inguinal lymph nodes were surgically removed from the mice, and then processed through mechanical disruption before digestion for 30 min at 37 °C in RPMI-1640 (10% heat-inactivated FBS and 1% PS) containing 1.5mg/mL collagenase IV(Sigma), 1.5mg/mL collagenase I (Sigma), 1.5 mg/mL hyaluronidase (Sigma), and 0.2 mg/mL DNase I (Sigma).<br>The samples were then passed through 200-mesh nylon mesh filters to obtain single-cell suspensions.<br>For tissue samples, the tissue was first mechanically disrupted from mice and divided into small pieces and homogenized in

	buffers to form single cell suspensions in the presence of digestive enzyme.<br>For all samples, cells were first stained with antibodies against surface antigens. In some experiments, cells were subsequently fixed, permeablized and stained for intracellular antigens.
Instrument	BD AccuritTM C6 Plus
Software	FlowJo X 10.0.7r2
Cell population abundance	No sorting was performed
Gating strategy	The detailed gating strategy could be found in method section.

☒ Tick this box to confirm that a figure exemplifying the gating strategy is provided in the Supplementary Information.
